# Analysis of protein missense alterations by combining sequence‐ and structure‐based methods

**DOI:** 10.1002/mgg3.1166

**Published:** 2020-02-25

**Authors:** Aram Gyulkhandanyan, Alireza R. Rezaie, Lubka Roumenina, Nathalie Lagarde, Veronique Fremeaux‐Bacchi, Maria A. Miteva, Bruno O. Villoutreix

**Affiliations:** ^1^ INSERM U973 Laboratory MTi University Paris Diderot Paris France; ^2^ Laboratory SABNP University of Evry INSERM U1204 Université Paris‐Saclay Evry France; ^3^ Cardiovascular Biology Research Program Oklahoma Medical Research Foundation Oklahoma City OK USA; ^4^ Department of Biochemistry and Molecular Biology University of Oklahoma Health Sciences Center Oklahoma City OK USA; ^5^ INSERM UMR_S 1138 Centre de Recherche des Cordeliers Paris France; ^6^ Sorbonne Universités Paris France; ^7^ Université Paris Descartes Sorbonne Paris Cité Paris France; ^8^ Laboratoire GBCM EA7528 Conservatoire national des arts et métiers Hesam Université Paris France; ^9^ Assistance Publique‐Hôpitaux de Paris Service d'Immunologie Biologique Hôpital Européen Georges Pompidou Paris France; ^10^ Inserm U1268 MCTR CNRS UMR 8038 CiTCoM Faculté de Pharmacie de Paris Univ. De Paris Paris France; ^11^ INSERM Institut Pasteur de Lille U1177‐Drugs and Molecules for Living Systems Université de Lille Lille France

**Keywords:** Antithrombin, CYP, Factor B, Factor VIII, missense variants, PolyPhen‐2, structural analysis, structural bioinformatics

## Abstract

**Background:**

Different types of in silico approaches can be used to predict the phenotypic consequence of missense variants. Such algorithms are often categorized as sequence based or structure based, when they necessitate 3D structural information. In addition, many other in silico tools, not dedicated to the analysis of variants, can be used to gain additional insights about the possible mechanisms at play.

**Methods:**

Here we applied different computational approaches to a set of 20 known missense variants present on different proteins (CYP, complement factor B, antithrombin and blood coagulation factor VIII). The tools that were used include fast computational approaches and web servers such as PolyPhen‐2, PopMusic, DUET, MaestroWeb, SAAFEC, Missense3D, VarSite, FlexPred, PredyFlexy, Clustal Omega, meta‐PPISP, FTMap, ClusPro, pyDock, PPM, RING, Cytoscape, and ChannelsDB.

**Results:**

We observe some conflicting results among the methods but, most of the time, the combination of several engines helped to clarify the potential impacts of the amino acid substitutions.

**Conclusion:**

Combining different computational approaches including some that were not developed to investigate missense variants help to predict the possible impact of the amino acid substitutions. Yet, when the modified residues are involved in a salt‐bridge, the tools tend to fail, even when the analysis is performed in 3D. Thus, interactive structural analysis with molecular graphics packages such as Chimera or PyMol or others are still needed to clarify automatic prediction.

## Graphical abstract

Combining different computational approaches help to investigate missense alterations. At present, it is still necessary to perform interactive structural analysis with molecular graphics systems to try to understand the potential impacts of the amino acid substitution.

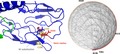

## 1 INTRODUCTION

Analysis of human genetic variations and its relationship to disease and drug response has gained remarkable attention these recent years. Genome‐wide association studies and candidate gene association studies tend to associate single nucleotide alterations with diseases. While these approaches are used at the DNA level, it is important to analyze, when possible, variations at the protein structural level as well. Structural analysis does indeed assist the development of rational hypotheses about possible impacts of substitutions and their possible links to disease states. Furthermore, if the protein under investigation is a therapeutic target, amino acid substitutions can cause drastic changes in drug target phenotypes, thereby resulting in dysfunctional drugs. Such investigations are therefore important in the field of drug discovery.

It is known that amino acid changes can affect both the function and the structure of a protein (i.e., alter catalysis, induce posttranslational modification, folding and stability, perturb ligand binding, favor multimerization, etc). Numerous studies have established the importance of combining experimental and in silico approaches for studying the influence of amino acid substitutions on the structure and function of proteins (Martiny & Miteva, 2013; Ramamoorthy & Skaar, 2011; Singh, Kashyap, Pandey, & Saini, 2011; Takano et al., 2012; Villoutreix, 2002; Witham, Takano, Schwartz, & Alexov, 2011). Nevertheless, it is not feasible to take an experimental approach for studying all the amino acid substitutions identified in patients' proteins. To assist the process and to gain knowledge about the possible impact of amino acid substitutions with the aim of reducing wet laboratory experiments, numerous bioinformatics methods have been developed (Kucukkal, Petukh, Li, & Alexov, 2015; Thusberg & Vihinen, 2009). These tools are usually categorized as sequence based, structure based, or involve the combination of both. The sequence‐based tools, in general, provide important information about conserved residues and can give some insights about how amino acid changes impact a protein. On the other hand, the second set of methods uses the 3D structures (experimental or high‐quality homology models) of the proteins, and the consensus belief/observation is that analyses performed at the structural levels are more reliable (Thusberg & Vihinen, 2009). Indeed, when 3D structures of proteins are known, several characteristics can be analyzed including stability and/or the dynamics of the mutant proteins and the impact of amino acid changes in different types of molecular interactions can be analyzed (Kucukkal et al., 2015). Yet, if numerous amino acid changes have to be analyzed, it is important to select fast 3D computational approaches. Overall, it is still difficult at present to decide which tools can reliably predict the pathogenic character of new variants (Grimm et al., 2015).

**Figure 1:**
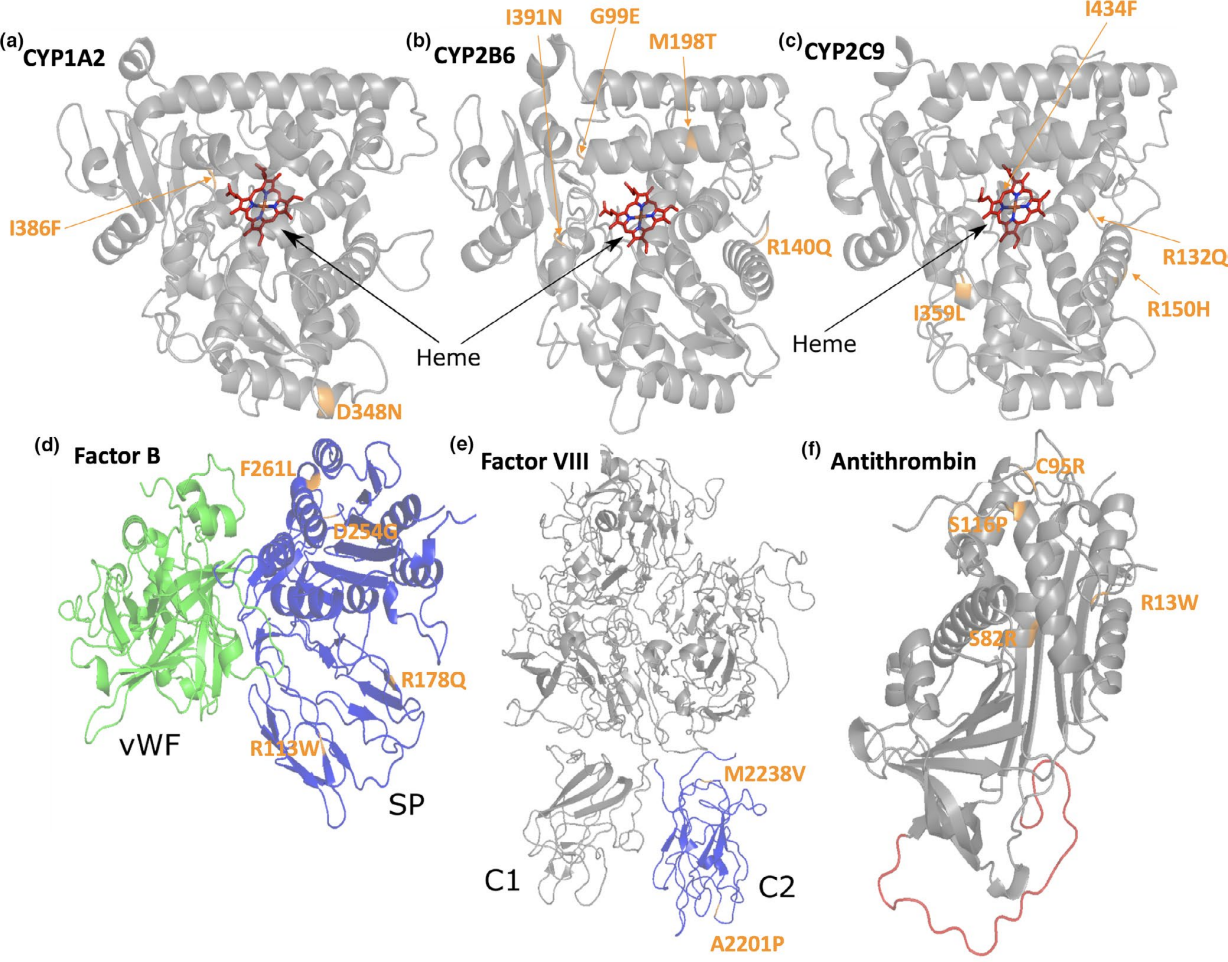
3D structures of the investigated proteins. The proteins are shown as cartoon and the substituted amino acids are highlighted in orange. A‐CYP 1A2 (Heme in red), B‐CYP 2B6 (Heme in red), C‐CYP 2C9 (Heme in red), D‐Complement Factor B (serine protease [SP] domain in blue and the von Willebrand factor [VWF] domain in green). E‐The factor VIII (FVIII) protein structure with the C2 domain colored in blue. F‐Antithrombin (AT) with the reactive center loop colored in red

In this study, we investigated 20 amino acid substitutions present in 6 different proteins involved in the health and disease states (Figure 1): (a) three members of the Cytochrome P450 (CYP) family (drug‐metabolizing enzymes responsible for the metabolism of most human drugs); (b) a protein from the complement system (a system involved in immune response and intended to protect the body from foreign agents); and (c) two proteins from the blood coagulation system.

The cytochrome P450 superfamily is responsible for the metabolism of about 90% of the common therapeutic drugs (Fujikura, Ingelman‐Sundberg, & Lauschke, 2015; Singh et al., 2011). Among human CYPs, the isoforms CYP1A2, CYP2C8, CYP2C9, CYP2C19, CYP2D6, CYP2E1, and CYP3A4 are responsible for the oxidative catalysis of most human drugs and many exhibit genetic polymorphisms (Fujikura et al., 2015; Singh et al., 2011). The genetic variations resulting in missense alterations can lead to loss‐of‐function variants or gain‐of‐function variants of CYP and can cause toxicity or lack of efficacy. Several CYP isoforms of the class 1 family are highly polymorphic (Daly, 2015; Martiny & Miteva, 2013; Zhou, Ingelman‐Sundberg, & Lauschke, 2017). Here, we have investigated missense alterations on CYP2B6, CYP2C9, and CYP1A2 proteins (Fujikura et al., 2015). The impact of missense alterations on the state of various CYP isoforms and their effect on drug metabolism has already been conducted by several research groups (Elfaki, Mir, Almutairi, & Duhier, 2018; Isvoran et al., 2017; Louet et al., 2018; Rydberg & Olsen, 2012; Simonetti et al., 2018) but in this article, we focused on a total of 10 missense alterations present on the CYP2B6, CYP2C9, and CYP1A2 isoforms. Of particular importance for the present study, CYP proteins have internal channels that play critical structural roles in the function of these proteins. These channels or tunnels tend to connect internal spaces of the proteins with the exterior surface, thereby enabling substrate/product transport toward the catalytic site. Some of these channels have been characterized, but there are still others that are not known.

The complement system proteins are key players in the innate immunity with over 20 protein components (Merle, Church, Church, Fremeaux‐Bacchi, & Roumenina, 2015; Merle, Noe, Noe, Halbwachs‐Mecarelli, Fremeaux‐Bacchi, & Roumenina, 2015). Factor B (FB) is a major component of the alternative pathway of complement activation. FB associates with the central complement component C3b to form the C3‐cleaving enzyme C3 convertase. The activity of this enzyme leads to the generation of novel derivatives C3, allowing the opsonization of pathogens, the generation of pro‐inflammatory anaphylatoxine C3a, and promoting the terminal membrane lytic complex of the complement pathway. Crystal structures of FB, of its fragments and domains (Ba, Bb, vWF‐A domain, and SP‐domain) and of the C3 convertase (C3bBb) have been reported, allowing detailed structural analyses of its functional properties (Bhattacharya, Lupher, Staunton, & Liddington, 2004; Forneris et al., 2010; Jing et al., 2000; Milder et al., 2007; Pedersen et al., 2017; Ponnuraj et al., 2004; Rooijakkers et al., 2009). Missense alterations causing changes in the FB function can have severe consequences, and in particular, lead to atypical hemolytic uremic syndrome (aHUS). aHUS is a rare genetic renal thrombotic microangiopathy disease, associated with genetic abnormalities and the over activation of the alternative complement pathway, causing a glomerular endothelial cells damage. Mutations in FB have been described in about 1% of the aHUS cases and extensive research toward understanding the functional consequences of specific mutations have been reported (Marinozzi et al., 2014). In the present study we investigated four selected amino acid substitutions in FB.

Blood coagulation comprises of more than 30 proteins that interact with each other with a high degree of specificity (Cardenas, Rein‐Smith, & Church, 2016; Palta, Saroa, & Palta, 2014; Versteeg, Heemskerk, Levi, & Reitsma, 2013; Villoutreix, 2002). Missense mutations identified in blood coagulation proteins can lead to life‐threatening illnesses. For example, amino acid changes in distinct regions of factor VIII (FVIII) can cause bleeding disorders (Hemophilia A) (Liu et al., 2000; Pratt et al., 1999; Spiegel, Jacquemin, Saint‐Remy, Stoddard, & Pratt, 2001). Of particular interest for the present study, some of the coagulation proteins including FVIII function properly only when they are anchored in an appropriate membrane surface. Another example is Antithrombin (AT). This plasma inhibitory protein is a key regulator of the coagulation system (Huntington, Olson, Fan, & Gettins, 1996; Izaguirre et al., 2014; Muszbek, Bereczky, Kovács, & Komáromi, 2010; Olson, Richard, Izaguirre, Schedin‐Weiss, & Gettins, 2010). This protein acts as an essential inhibitor of activated factor X (Xa) and thrombin (IIa), and a number of other activated coagulation factors (Abildgaard, 2007; Huntington et al., 1996; Izaguirre et al., 2014; Muszbek et al., 2010; Olson et al., 2010). AT becomes an effective inhibitor only upon binding to heparin or heparan sulfate proteoglycans lining the vascular endothelium (Cooper, Coath, Daly, & Makris, 2011; Huntington et al., 1996). Numerous alterations have been identified in the antithrombin gene, which are mainly missense alterations (Bayton & Lane, 2^2003^; Luxembourg et al., 2011; Stenson et al., 2014). In the present study, we investigated two amino acid substitutions in FVIII and four in antithrombin.

The goal of this article is to illustrate how 2D and 3D in silico analysis of amino acid changes can complement each other in developing hypotheses about the possible impacts of the substitutions on the structure and function of the investigated proteins and also highlight their limitations. Furthermore, we suggest that it can be valuable to also use in silico methods not dedicated to the prediction of missense alterations. In addition, we wanted to investigate the benefit of using automatic 3D mapping online services and if 3D interactive analysis of the amino acid substitution using molecular graphics packages could provide additional insights. We have selected 20 illustrative amino acid changes with associated experimental/clinical data. Clearly, there are numerous in silico tools that can be used to explore the possible impact of amino acid changes on the structure and function of a protein (Hu et al., 2019; Villoutreix, Lagorce, Labbé, Sperandio, & Miteva, 2013), and here we have used a few diverse, fast, relatively recent and user‐friendly sequence‐based and structure‐based computational approaches (see Table 1).

**Table 1:** In silico tools and databases used in the present study

Tool	URL	Input
PolyPhen‐2 (investigation of variants)	http://genetics.bwh.harvard.edu/pph2	Sequence
PopMusic (ΔΔG)	http://dezyme.com	Uploaded 3D structure
DUET (ΔΔG)	http://biosig.unimelb.edu.au/duet	Uploaded 3D structure
MaestroWeb (ΔΔG)	https://biwww.che.sbg.ac.at/maestro/web	Uploaded 3D structure
SAAFEC (ΔΔG)	http://compbio.clemson.edu/SAAFEC	Uploaded 3D structure
Missense3D	http://www.sbg.bio.ic.ac.uk/~missense3d	Protein sequence or 3D structure/PDB ID
VarSite	https://www.ebi.ac.uk/thornton-srv/databases/VarSite	UniProt ID, or search terms or disease
FlexPred (flexibility prediction)	http://kiharalab.org/flexPred	Uploaded 3D structure
PredyFlexy (flexibility prediction)	http://www.dsimb.inserm.fr/dsimb_tools/predyflexy	Sequence
Clustal Omega (multiple sequence alignment)	https://www.ebi.ac.uk/Tools/msa	Sequence
meta‐PPISP (prediction of PPI sites)	http://pipe.scs.fsu.edu/meta-ppisp	Uploaded 3D structure
FTMap (binding pocket prediction)	http://ftmap.bu.edu/home.php	Uploaded 3D structure
ClusPro (macromolecular docking)	https://cluspro.org	Uploaded 3D structure
PPM (protein membrane interaction prediction)	http://opm.phar.umich.edu	Uploaded 3D structure
pyDockWeb (macromolecular docking)	https://life.bsc.es/pid/pydock	Uploaded 3D structure
RING‐2.0 (residue interaction network)	http://protein.bio.unipd.it/ring	Uploaded 3D structure
Cytoscape (data visualization)	http://www.cytoscape.org	RING output XLM file
ChannelsDB (investigate channels)	https://webchemdev.ncbr.muni.cz/ChannelsDB	PDB ID
PharmVar (variation database)	https://www.pharmvar.org/htdocs/archive/index_original.htm	—
gnomAD (Genome Aggregation Database)	https://gnomad.broadinstitute.org/	—
ClinVar (genomic variation database)	https://www.ncbi.nlm.nih.gov/clinvar/	—
Chimera (molecular graphics)	http://www.cgl.ucsf.edu/chimera	—
PyMol (molecular graphics)	http://www.pymol.org	—
PDB (protein structure database)	https://www.rcsb.org	—
UniProt (annotated protein database)	http://www.uniprot.org	—

## 2 METHODS

Ethical Compliance. The manuscript is a retrospective case report that does not require ethics committee approval.

Tools and key databases that were used in this study are reported in Table 1.

### 2.1 Protein structures and variant databases

The following experimental structures were downloaded from the Protein Data Bank (PDB) (Berman et al., 2000) and used in our study: CYP 1A2 (PDB ID: http://www.rcsb.org/pdb/search/structidSearch.do?structureId=2HI4) (Sansen et al., 2007), CYP 2B6 (PDB ID: http://www.rcsb.org/pdb/search/structidSearch.do?structureId=3IBD) (Shah et al., 2010), CYP 2C9 (PDB ID: http://www.rcsb.org/pdb/search/structidSearch.do?structureId=1OG2) (Williams et al., 2003), complement factor B (PDB ID: http://www.rcsb.org/pdb/search/structidSearch.do?structureId=2OK5) (Milder et al., 2007), coagulation factor VIII (PDB ID: http://www.rcsb.org/pdb/search/structidSearch.do?structureId=2R7E) (Shen et al., 2008), and antithrombin (PDB ID: http://www.rcsb.org/pdb/search/structidSearch.do?structureId=2BEH) (Johnson et al., 2006). Heteroatoms were removed except for the heme group of the CYP proteins. Earlier, the nomenclature and properties of allelic variants of human CYP could be found at http://www.cypalleles.ki.se (Sim & Ingelman‐Sundberg, 2013). However, the Human Cytochrome P450 Allele Nomenclature Database can now be found at the Pharmacogene Variation (PharmVar) Consortium (Gaedigk et al., 2018). Thus, all investigated amino acid changes for the three types of CYP proteins were taken from the PharmVar updated list. Some missing side chains or engineered residues present in the experimental structures were added or changed back to the wild‐type sequence with the Chimera software (Pettersen et al., 2004). The PyMOL Molecular Graphics System (Version 1.8.2.2 Schrödinger, LLC) and Chimera were both used for the structural analysis.

### 2.2 Computational approaches and databases

#### 2.2.1 PolyPhen‐2

We selected PolyPhen‐2 (*Poly*morphism *Phen*otyping) (Adzhubei et al., 2010) to investigate the amino acid changes because this method is fast, user‐friendly and used in many laboratories. The tool is mainly sequence based but it also incorporates some structural information when available to produces qualitative predictions. In the current 2.2 version, the mutations are labeled as "benign," "possibly damaging," and as "probably damaging."

#### 2.2.2 Protein stability prediction in 3D

For the assessment of the protein stability, we used four different programs: PopMusic, DUET, MaestroWeb, and SAAFEC. These web servers compute ΔΔG values between the wild‐type and the variant proteins. *PoPMuSiC* (Gonnelli, Rooman, & Dehouck, 2012) is a software which evaluates the changes in the stability of a given protein upon amino acid changes. The server predicts the thermodynamic stability changes caused by a single site substitution using a linear combination of statistical potentials whose coefficients depend on the solvent accessibility of the modified residue (Dehouck, Kwasigroch, Gilis, & Rooman, 2011). *DUET* is a software that evaluates protein stability with an optimized predictor that makes use of support vector machine approaches (Pires, Ascher, & Blundell, 2014aa). DUET consolidates two complementary approaches SDM (Pandurangan, Ochoa‐Montaño, Ascher, & Blundell, 2017) and mCSM (Pires, Ascher, & Blundell, 2014bb) in a consensus prediction. This is achieved by combining the results of the two separate methods with an optimized predictor. *MAESTRO* is a method for predicting changes in stability. It is a structure‐based approach that provides predicted free energy change (ΔΔG) values as well as corresponding confidence estimation values for the predictions while at the same time allowing for high‐throughput scanning of multi‐point amino acid changes (Laimer, Hiebl‐Flach, Lengauer, & Lackner, 2016; Laimer, Hofer, Fritz, Wegenkittl, & Lackner, 2015). The *SAAFEC* (Single Amino Acid Folding Free Energy Changes) method (Getov, Petukh, & Alexov, 2016) is designed for calculating the folding free energy changes caused by missense alterations. Based on the MM‐PBSA method (Homeyer & Gohlke, 2012) with weight coefficients, this approach was optimized using experimental data from the ProTherm database (Bava, Gromiha, Uedaira, Kitajima, & Sarai, 2004). With this approach, the protein structures undergo an energy minimization using the NAMD software (Phillips et al., 2005).

#### 2.2.3 Automatic 3D online structural mapping of missense variants

Two web servers were used: Missense3D (Ittisoponpisan et al., 2019) and VarSite (Laskowski, Stephenson, Sillitoe, Orengo, & Thornton, 2020). The *Missense3D* pipeline maps and analyze amino acid changes on experimental and homology model protein 3D structures. *VarSite* maps known disease‐associated variants from UniProt (UniProt Consortium, 2019), ClinVar (Landrum et al., 2014), and gnomAD (genome aggregation database) (Karczewski et al., 2019) or data provided by the users onto protein experimental 3D structures. A disease propensity score is also reported, the value quantifies how much more often a variant is observed in diseases than in the natural variant data obtained from gnomAD. The value ranges from very low (propensity = 0.25) to very high (propensity = 3.27). On both servers, users obtain a report card with information about the amino acid substitution.

#### 2.2.4 Multiple sequence alignment

To investigate sequence conservation for the abovementioned proteins, multiple sequence alignments (MSA) were performed with the EMBL‐EBI *Clustal Omega* server (Sievers et al., 2011) using as input sequences from different species downloaded from the UniProtKB database (UniProt Consortium, 2019).

#### 2.2.5 Protein flexibility

Some regions of proteins can be moderately to highly flexible. Flexibility can be inferred in some cases from X‐ray experiments, obtained from NMR studies or explored using long molecular simulation approaches. Yet, some very fast approaches have been reported to provide relatively accurate predictions without a need for CPU/GPU intensive calculations. We here employed the predicted B‐factor (relative vibrational motion) and RMSFs (root‐mean‐square fluctuations) obtained from the prediction program *PredyFlexy* (fast computations carried out only over the protein sequences) (de Brevern, Bornot, Craveur, Etchebest, & Gelly, 2012). Three types of flexibility are proposed by this approach. PredyFlexy classifies amino acid residues into rigid, intermediate or flexible sites. We also used the program *FlexPred*, a fast method that uses the protein 3D structure as input and predicting fluctuations using easily computed static structural features (Kuznetsov & McDuffie, 2008). Overall, the tool determines which amino acid residues are located in flexible sites or in more rigid regions (Jamroz, Kolinski, & Kihara, 2012; Kuznetsov & McDuffie, 2008).

#### 2.2.6 Residue interaction network and visualization

The Residue Interaction Network Generator (RING) software was used to gain additional insights into the structures of the selected proteins through visualization of nonbonded interactions (Martin et al., 2011). The *RING‐2.0* server was used in our study (Piovesan, Minervini, & Tosatto, 2016). The generated RING network files were analyzed with *Cytoscape*, a tool that provides a basic set of features for data integration, analysis, and visualization (Shannon et al., 2003).

#### 2.2.7 Predictions of ligand‐binding pockets

To investigate binding pockets and ligand‐binding hot spots we used the *FTMap* fragment mapping server (Brenke et al., 2009; Kozakov et al., 2015, 2011; Ngan et al., 2012). FTMap positions 16 small organic probe molecules of varying size, shape, and polarity over the protein surface to predict binding pockets and key residues involved. This approach can also provide insights into protein–protein interface regions.

#### 2.2.8 Protein–Protein interaction site prediction

To predict protein–protein interaction sites, we used the *meta‐PPISP* web server (Qin & Zhou, 2007). A number of complementary methods have been developed for predicting protein–protein interaction sites. By combining results from different predictors, the meta‐PPISP method was found to improve prediction robustness and accuracy. Its operating system is built on three different web servers: cons‐PPISP (Chen & Zhou, 2005; Zhou & Shan, 2001), PINUP (Liang, Zhang, Liu, & Zhou, 2006), and Promate (Neuvirth, Raz, & Schreiber, 2004).

#### 2.2.9 Channel‐related analyses for the CYP proteins

For these investigations we used the *ChannelsDB* database (Pravda et al., 2018). Channels, tunnels, and pores are very important structural features within biomacromolecules. Tunnels connect internal spaces with exterior regions. Enzyme active sites can be connected to the exterior environment by one or more channels passing through the protein (Louet et al., 2018; Pravda et al., 2014). Here, CYP proteins were investigated with such tools.

#### 2.2.10 Antibody–protein and heparin–protein docking

Protein–protein and heparin–protein docking computations were carried out with the *ClusPro* server. ClusPro offers a number of options for docking experiments in particular modes tuned for antibody and heparin molecules (Kozakov et al., 2017; Mottarella et al., 2014). To study the interaction of an antibody with the FVIII C2 domain the ClusPro server was used (Brenke et al., 2012). Additional docking experiments between a Fab fragment and FVIII C2 domain were performed with the *pyDockWeb* server (Jiménez‐García, Pons, & Fernandez‐Recio, 2013). Docking of heparin on antithrombin was performed using the advanced options of the ClusPro server (Kozakov et al., 2017; Mottarella et al., 2014).

#### 2.2.11 Protein–membrane interaction prediction

To position FVIII at the surface of a membrane, we used the *PPM* web server (Lomize, Pogozheva, Joo, Mosberg, & Lomize, 2012). The location and orientation of the protein are obtained by various rotations and translations with the goal of optimizing the protein transfer energy from water to a virtual lipid bilayer.

## 3 RESULTS AND DISCUSSION

We investigated several CYP450, complement, and blood coagulation proteins. To analyze the potential impacts of the selected amino acid changes, we first used PolyPhen‐2 and then additional sequence‐ and structure‐based approaches associated with other structural bioinformatics tools not developed specifically to investigate point mutations and with interactive 3D structural analysis. Also, two automatic 3D mapping tools (Missense3D, VarSite) were used to gather additional information about the possible impact of the amino acid substitutions and to compare the outputs with the 3D interactive investigations carried out with standalone molecular graphics engine PyMol and Chimera. Our analysis focuses on highlighting the differences and complementarity of the in silico methods as well as limitations. Warnings are reported based on our observations together with some recommendations.

The mainly sequence‐based approach PolyPhen‐2 was used with other structure‐based approaches (DUET, PopMusic, SAAFEC, MAESTROweb). Different computations not fully dedicated to the study of amino acid substitutions were also used. Residue interaction network computation is used to gain knowledge about protein structures and functions. In this context, amino acid residues are referred to as nodes while edges represent noncovalent interactions. Structural features can be reported for each node such as degree, here the number of noncovalent interactions with surrounding amino acids or cofactors. Intuitively, a substitution that involves a highly connected residue is likely to perturb the structure and/or the function of the protein. We used the RING sever (Martin et al., 2011; Piovesan et al., 2016) to carry out such investigation followed by visualization performed with Cytoscape (Shannon et al., 2003). It is also important to estimate whether the amino acid change is located in a very rigid area or in a flexible region since this can change the dynamics of the system. When dealing with several substitutions, obviously the use of fast computational approaches is recommended. For these reasons we decided to use the structure‐based FlexPred approach (Kuznetsov & McDuffie, 2008) as well as the sequence‐based PredyFlexy tool (de Brevern et al., 2012). Damaging amino acid changes are often located in the core interior of proteins, but it is also known that when they are solvent exposed, damaging amino acid substitutions tend to be located in protein–protein interaction sites (Yates & Sternberg, 2013). We used the meta‐PPISP web server (Qin & Zhou, 2007) to predict such regions. Other types of information can be investigated in silico, depending on the molecular functions that need to be addressed, via for instance, protein–protein docking or protein–heparin docking experiments, prediction of putative ligand‐binding sites or of channels, or predictions of zones potentially important for membrane interactions. Of importance, we note that many laboratories working with amino acid substitutions worldwide make little use of 3D structural data, we investigated the benefit of using automatic 3D mapping tools such as VarSite and Missense3D.

### 3.1 CYP1A2

For CYP1A2 (PDB ID: http://www.rcsb.org/pdb/search/structidSearch.do?structureId=2HI4, UniProt: http://www.uniprot.org/uniprot/P05177), we investigated the possible molecular effects of two clinically important alterations (Asp348Asn and Ile386Phe) (Table 2). Human cytochrome P450 1A2 catalyzes important reactions during the metabolism of xenobiotics, including N‐hydroxylation of carcinogenic aromatic amines. Variants Asp348Asn and Ile386Phe were expressed at levels less than half of the wild‐type proteins and these differences (vs. wild‐type) were statistically significant (*p* < 0.05) (Zhou, Josephy, Kim, & Guengerich, 2004). Table 2 shows results of the in silico analyses for CYP1A2 (PDB ID: http://www.rcsb.org/pdb/search/structidSearch.do?structureId=2HI4). CYP1A2 is not processed yet on the ChannelsDB server. Yet, from our previous work, it seems possible that Ile386 is located in one of the substrate‐binding channel (see Figure 6, channel 2f in Louet et al., 2018). Potential ligand‐binding pockets were also investigated with FTMap (Brenke et al., 2009; Kozakov et al., 2015, 2011; Ngan et al., 2012) and 14 binding pockets were suggested (Table S1). Only the amino acid Ile386 is predicted to be located in a ligand‐binding pocket (Table 2).

**Table 2:** In silico analysis of CYP1A2 (PDB ID: http://www.rcsb.org/pdb/search/structidSearch.do?structureId=2HI4, UniProt: http://www.uniprot.org/uniprot/P05177; GenBank: NM_000761.5)

CYP1A2 Amino acid/mutation/alleles	DUET ΔΔG, kcal/mol	PopMusic^a^ ΔΔG, kcal/mol	SAAFEC ΔΔG, kcal/mol	MAESTROweb^b^ ΔΔG, kcal/mol	PolyPhen‐2 Score/mutation prediction	MSA^c^ aa conservation	Involved in a predicted ligand‐binding pockets/pocket No. (FTMap)	Involved in known or predicted channel	Involved in predicted PPI sites (meta‐PPISP)	Node degree (RING‐2.0)	Predicted fluctuation value (FlexPred)	Flexibility class^d^ (PredyFlexy)	Decreased metabolic activity or protein expression	References
**Asp348** **D348N** CYP1A2*3	−0.297 Destabilizing	0.15 Destabilizing SA = 48.0%	−1.925 Destabilizing	−0.216 Stabilizing Cpred = 0.903	0.053 Benign	High	No	No	No	8	1.831	0	D348N was expressed at levels less than half of the wild type (in vitro)	Zhou et al. (2004)
**Ile386** **I386F** CYP1A2*4	−1.311 Destabilizing	0.98 Destabilizing SA = 13.5%	−0.283 Destabilizing	−0.039 Stabilizing Cpred = 0.937	0.998 Probably damaging	High	Yes/pockets 1 & 3 & 8	Yes	Yes	7	1.252	1	I386F was expressed at levels less than half of the wild type (in vitro)	Zhou et al. (2004)

a For the program PopMusic solvent accessibility (SA) values are shown (in percent).

b For the program MaestroWeb, the confidence estimation Cpred is shown (0.0‐not reliable and 1.0‐highly reliable).

c MSA‐Multiple sequence alignment.

d Flexibility class was determined by the program PredyFlexy (rigid‐0, intermediate‐1, flexible‐2).

Wild‐type residue (bold) and amino acid substitution (Underlined).

#### 3.1.1 Asp348Asn (allele CYP1A2*3)

The PolyPhen‐2 result suggests that this change is benign but three structure‐based tools indicate that the substitution is moderately destabilizing (specially SAAFEC) while MaestroWeb proposes a small stabilization effect. Asp348 is strictly conserved in our multiple sequence alignment (Figure S1) and partially solvent exposed (SA = 48.03%), located on a helical structure (Figure 1) and predicted to be in a relatively rigid area of the protein. It forms a salt‐bridge with Arg353 that will be lost upon its replacement with an asparagine (data not shown). Possibly, this substitution destabilizes this region of the protein. This residue makes several additional noncovalent interactions with its surrounding (node degree = 8), it is not thought to be part of a ligand‐binding pocket or a channel (Louet et al., 2018), and it is not predicted to be involved in a protein–protein interaction site (Table 2). Overall, three of the four 3D web servers suggest that this substitution could be problematic, in overall agreement with our 3D interactive structural analysis suggesting a possible destabilizing effect for this substitution. This in turn is in agreement with the in vitro experimental data since it was found that the expression level of the mutant was lower than that of the wild type, while the activity level of the mutant was also decreased (Zhou et al., 2004). Missense3D, reports that no structural damage is detected for this substitution (i.e., the salt‐bridge, about 3.6 Å between the charged groups, is not found) while VarSite underlines that the residue is highly conserved (in 110 aligned sequences), that the protein function could be perturbed and that there are not natural variants recorded in the gnomAD database at this position. The disease propensity score to the Asp to Asn change is in the scale of the method considered as low (value = 0.99). Taken together, for CYP1A2 Asp348Asn, the PolyPhen‐2 prediction is not supported by the ΔΔG stability studies nor with the interactive analyses performed with the PyMol/Chimera molecular graphics systems. The 3D analyses tend to support the experimental data and provide some hypotheses about the possible mechanisms resulting from the amino acid change. The automatic mapping Missense3D method does not flag this substitution while VarSite cautions about a possible impact on the structure/function of the modified protein.

#### 3.1.2 Ile386Phe (allele CYP1A2*4)

The PolyPhen‐2 data suggest that this substitution is most likely damaging, in agreement with three ΔΔG structure‐based approaches (except for MaestroWeb that suggests more a neutral effect) (Table 2). This residue is strictly conserved in our multiple sequence alignment (Figure S1), it is located in a loop and is mainly buried (SA = 13.52%) in a tightly packed hydrophobic and aromatic environment with several noncovalent interactions with its surrounding (node degree = 7) while being close to the Heme group (about 4 Å, Figure 1). It is predicted to be in a relatively rigid area. The residue is located in the catalytic pocket and in the vicinity of the substrate access channel. Interactive analysis suggests that the Ile to Phe substitution should generate steric clashes, possibly locally destabilize the protein and impair the catalytic activity. Missense3D results do not highlight structural damage while VarSite underlines an aliphatic to aromatic substitution, that the residue is highly conserved (in 111 aligned sequences) and that the protein function could be perturbed as residue 386 has contact with a ligand in several CYP 3D structures. The disease propensity value (=1.13) is in this case labeled as high. For CYP1A2 Ile386Phe, the most likely damaging effect predicted by PolyPhen‐2 is in good agreement with most 3D ΔΔG investigations and with our interactive 3D studies, further highlighted by the VarSite report card but not by the Missense3D results. Overall, most in silico investigations are in good agreement with the in vitro experimental observations, all supporting the basis for a decreased level of expression of the mutant protein (Chevalier et al., 2001; Zhou et al., 2004).

### 3.2 CYP2B6

For CYP2B6 (PDB ID: http://www.rcsb.org/pdb/search/structidSearch.do?structureId=3IBD, UniProt: http://www.uniprot.org/uniprot/P20813), we investigated the possible molecular effect of four clinically important alterations (Table 3). The drugs metabolized by CYP2B6 include the prodrug cyclophosphamide, the antimalarial artemisinin, the anesthetics ketamine and propofol, and the HIV‐1 reverse transcriptase inhibitors nevirapine and efavirenz (Lang et al., 2004). In vitro studies revealed that these CYP2B6 protein variants (Arg140Gln, Gly99Glu, and Ile391Asn) have a reduced expression level and/or activity. Two amino acid changes (Gly99Glu and Ile391Asn) resulted in almost undetectable enzyme activity, despite the presence of residual protein level (Lang et al., 2004). Resequencing defined loss‐of‐function allele *27 (Met198Thr), resulted in 85% decrease in enzyme activity (Rotger et al., 2007).

**Table 3:** In silico analysis of CYP2B6 (PDB ID: http://www.rcsb.org/pdb/search/structidSearch.do?structureId=3IBD, UniProt: http://www.uniprot.org/uniprot/P20813; GenBank: NM_000767.5)

CYP2B6 Amino acid/mutation/alleles	DUET ΔΔG, kcal/mol	PopMusic^a^ ΔΔG, kcal/mol	SAAFEC ΔΔG, kcal/mol	MAESTROweb^b^ ΔΔG, kcal/mol	PolyPhen‐2 Score/mutation prediction	MSA^c^ aa conservation	Involved in predicted ligand‐binding pockets/pocket No. (FTMap)	Involved in predicted or known channels	Involved in predicted PPI sites (meta‐PPISP)	Node degree (RING‐2.0)	Predicted fluctuation value (FlexPred)	Flexi‐bility class^d^ (PredyFlexy)	Decreased metabolic activity or protein expression	References
**Gly99** **G99E** CYP2B6*12	−2.245 Destabilizing	1.49 Destabilizing SA = 3.3%	−0.491 Destabilizing	0.025 Destabilizing Cpred = 0.924	1.000 Probably damaging	High	No	Yes	No	3	1.052	1	Amino acid changes resulted in almost undetectable enzyme activity (in vitro)	Lang et al. (2004)
**Arg140** **R140Q** CYP2B6*14	−0.562 Destabilizing	0.32 Destabilizing SA = 40.9%	−3.798 Destabilizing	0.471 Destabilizing Cpred = 0.786	0.020 Benign	High	Yes/pocket 9	No	Yes	7	1.747	1	Reduced expression and/or function activity of protein (in vitro)	Lang et al. (2004)
**Met198** **M198T** CYP2B6*27	−1.374 Destabilizing	1.12 Destabilizing SA = 26.2%	−0.580 Destabilizing	−0.278 Stabilizing Cpred = 0.832	0.000 Benign	High	Yes/pocket 1	No	Yes	10	1.223	0	Loss‐of‐function allele *27 results in 85% decrease in enzyme activity.	Rotger et al. (2007)
**Ile391** **I391N** CYP2B6*15	−3.149 Destabilizing	2.71 Destabilizing SA = 0.0%	−1.012 Destabilizing	0.992 Destabilizing Cpred = 0.890	1.000 Probably damaging	High	No	No	No	11	0.897	1	Amino acid changes resulted in almost undetectable enzyme activity (in vitro)	Lang et al. (2004)

a For the program PopMusic solvent accessibility (SA) values are shown (in percent).

b For the program MaestroWeb the confidence estimation Cpred is shown (0.0‐not reliable and 1.0‐highly reliable).

c MSA‐Multiple sequence alignment.

d Flexibility class was determined by the program PredyFlexy (rigid‐0, intermediate‐1, flexible‐2).

Wild‐type residue (bold).

The results of our computations are shown in Table 3. Four channels were found for the CYP2B6 protein with ChannelsDB (Pravda et al., 2018). From the four amino acids investigated here, only Gly99 seems to be involved in forming a channel. We predicted 19 ligand‐binding pockets with the FTMap server (Brenke et al., 2009; Kozakov et al., 2015, 2011; Ngan et al., 2012) and Arg140 and Met198 belong to the predicted binding pocket numbers 9 and 1, respectively (Table S2).

#### 3.2.1 Gly99Glu (allele CYP2B6*12)

**Figure 2:**
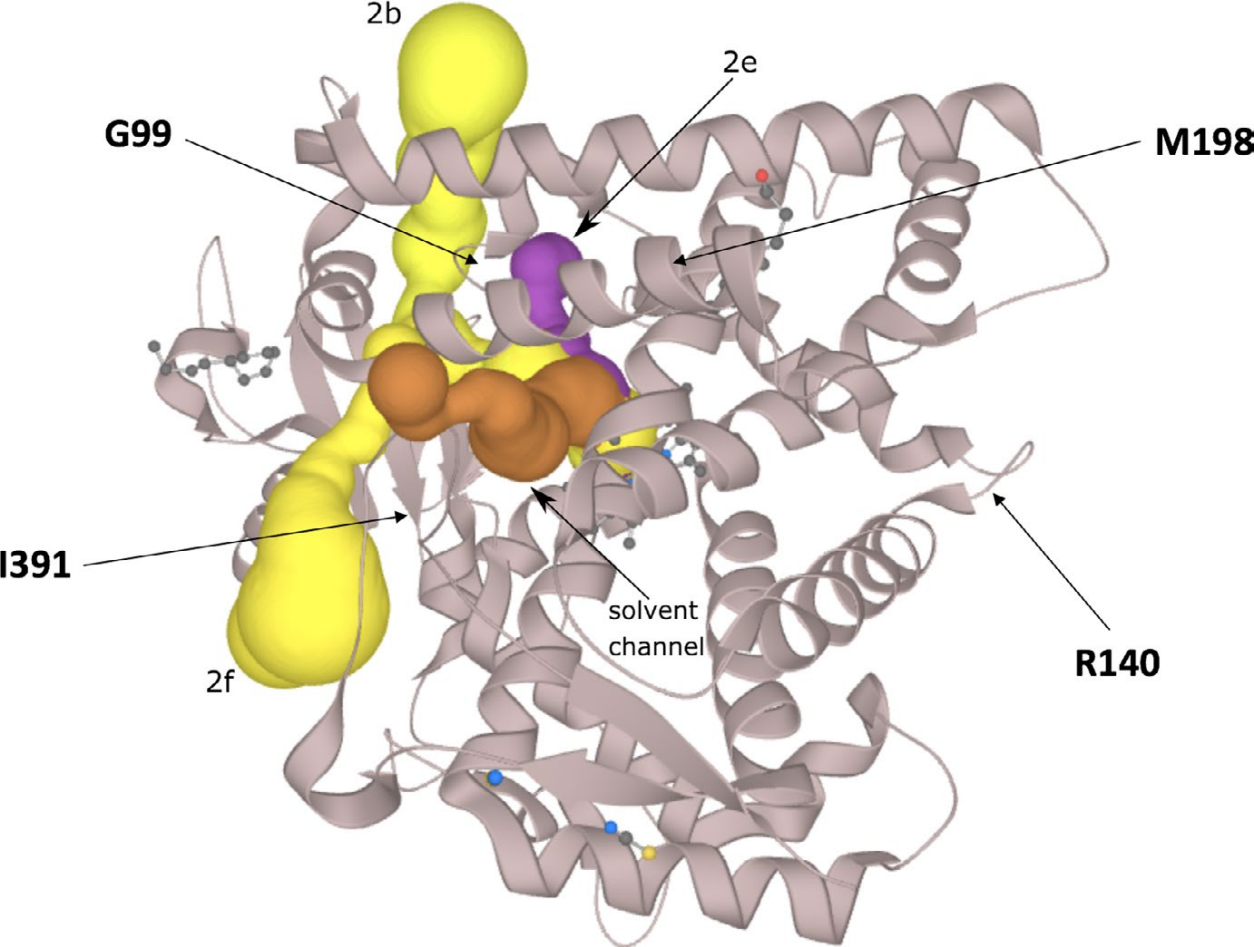
Overview of the CYP2B6 protein (PDB ID: http://www.rcsb.org/pdb/search/structidSearch.do?structureId=3IBD) with the predicted channels 2e (magenta, solid surface), 2b and 2f (yellow, solid surface). A solvent channel is shown in brown (solid surface)

PolyPhen‐2 suggests that this amino acid change is most likely damaging. This conclusion matches the results obtained with three structure‐based programs that predict a destabilizing effect (MAESTROweb suggests more a neutral effect). This residue is buried (SA = 3.29%) and relatively close to the Heme group (Figure 1). It is fully conserved in our MSA (Figure S2) and part of the channel 2e (Figure 2) but it is not predicted to be part of a putative ligand‐binding pocket nor is present in a putative PPI site. There is no room to accommodate a glutamate in this region of the protein and the amino acid change would in addition possibly put the substituted residue next to a negatively charged residue, Glu387. Most in silico results suggest that this substitution could be destabilizing in agreement with the in vitro experiments in which the amino acid change resulted in an almost undetectable enzyme activity (Lang et al., 2004). The automatic 3D mapping tool, Missense3D, reports that the substitution replaces a buried uncharged and small residue by a large and charged one while VarSite underlines a large difference in term of amino acid substitution and a likely change in the protein's function (the residue is found highly conserved in about 175 protein sequences). The disease propensity score is high with a value of 1.67. For CYP2B6 Gly99Glu, essentially all the silico tools tend to be in good agreement and assist the analysis of the experimental studies.

#### 3.2.2 Arg140Gln (allele CYP2B6*14)

**Figure 3:**
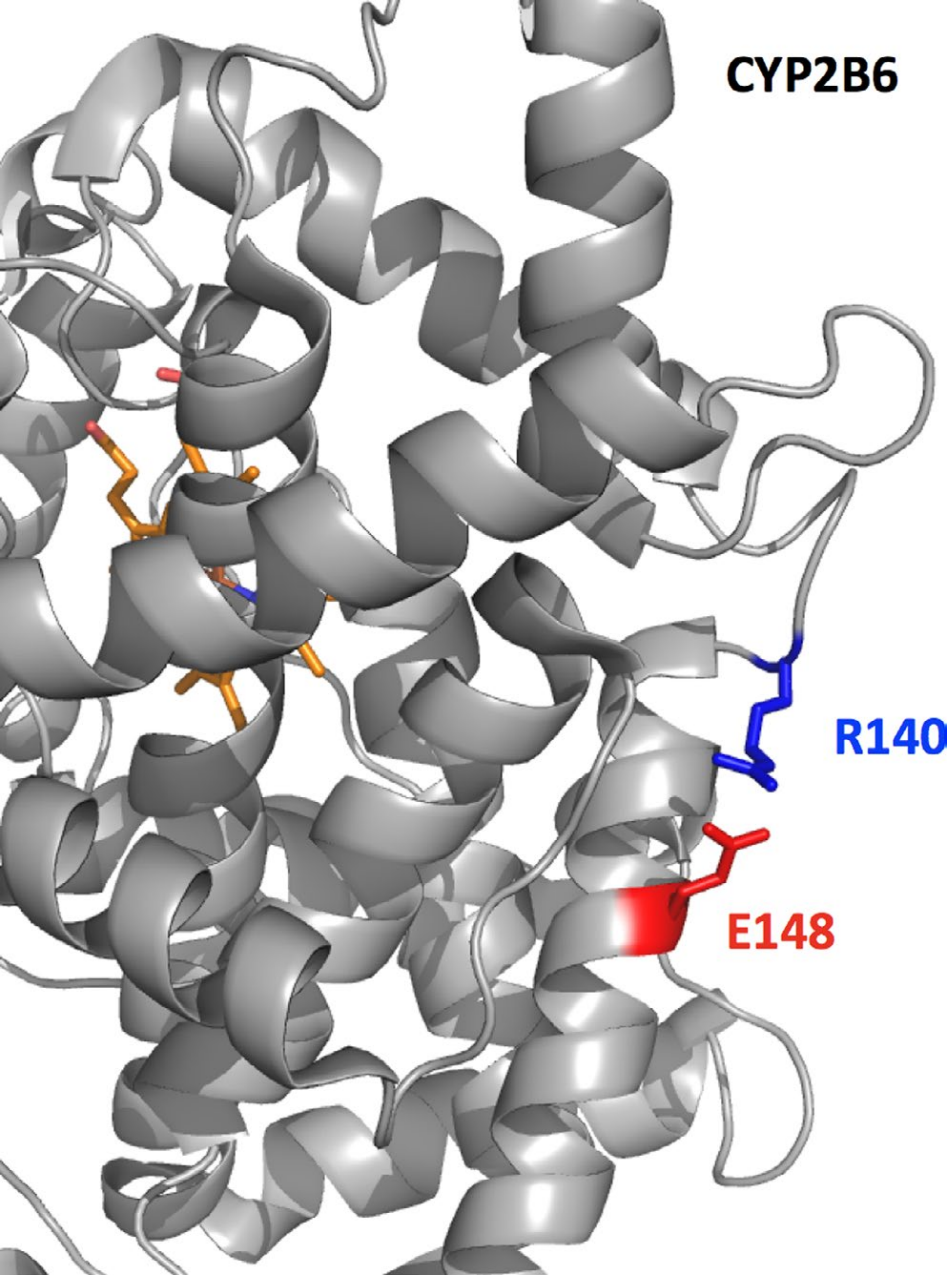
Part of CYP2B6 (PDB ID: http://www.rcsb.org/pdb/search/structidSearch.do?structureId=3IBD, cartoon representation) is presented with a zoom on residue Arg140 (blue). The protein is in gray and the heme (part of the catalytic site) in red‐orange. Arg140 is partially solvent exposed, forming a salt‐bridge (~3.2 Å) with Glu148 (red)

PolyPhen‐2 suggests that this mutation is benign while all the structural approaches propose more a destabilizing effect. This residue is not in the catalytic pocket and is in part solvent exposed (SA = 41.28%) (Figure 1). It forms a salt‐bridge with Glu148 (Figure 3). This residue is fully conserved in the MSA (Figure S2) underlining its likely importance. Arg140 is located in a slightly flexible region (Table 3; Figure S3). The residue is predicted to be involved in a PPI interaction site (Figure S4) and indeed it may be involved in electrostatic interaction with the P450 oxidoreductase protein (POR) (Lee et al., 2014). It is not predicted to be involved in a channel (Table 3). The residue R140 has several noncovalent interactions with its surrounding (node degree = 7) (Figure 2). Thus, this substitution would likely damage the protein structure and function as predicted by most of the in silico tools used with the exception of PolyPhen‐2. The predictions of the structure‐based approaches are in agreement with the experimental data regarding the reduced expression and/or activity of the mutant protein (in vitro) (Lang et al., 2004). Missense3D, reports no structural damage (the salt‐bridge is not found but clearly visible in Figure 3, the distance between the charged groups is about 3.2 Å) while VarSite results underline that the substitution is somewhat conservative but suggest a likely change in the protein's function (the residue is found highly conserved in about 190 protein sequences). Two natural variants at this position are known (Trp and Pro). The disease propensity score is high with a value of 1.19. Residue Arg140 is also reported by VarSite as involved in PPI. For the CYP2B6 Arg140Gln, most 3D in silico outputs tend to be in good agreement with the experimental studies and help to propose possible molecular mechanisms associated with the substitution. These 3D results are not in agreement with the PolyPhen‐2 prediction.

#### 3.2.3 Met198Thr (allele CYP2B6*27)

**Figure 4:**
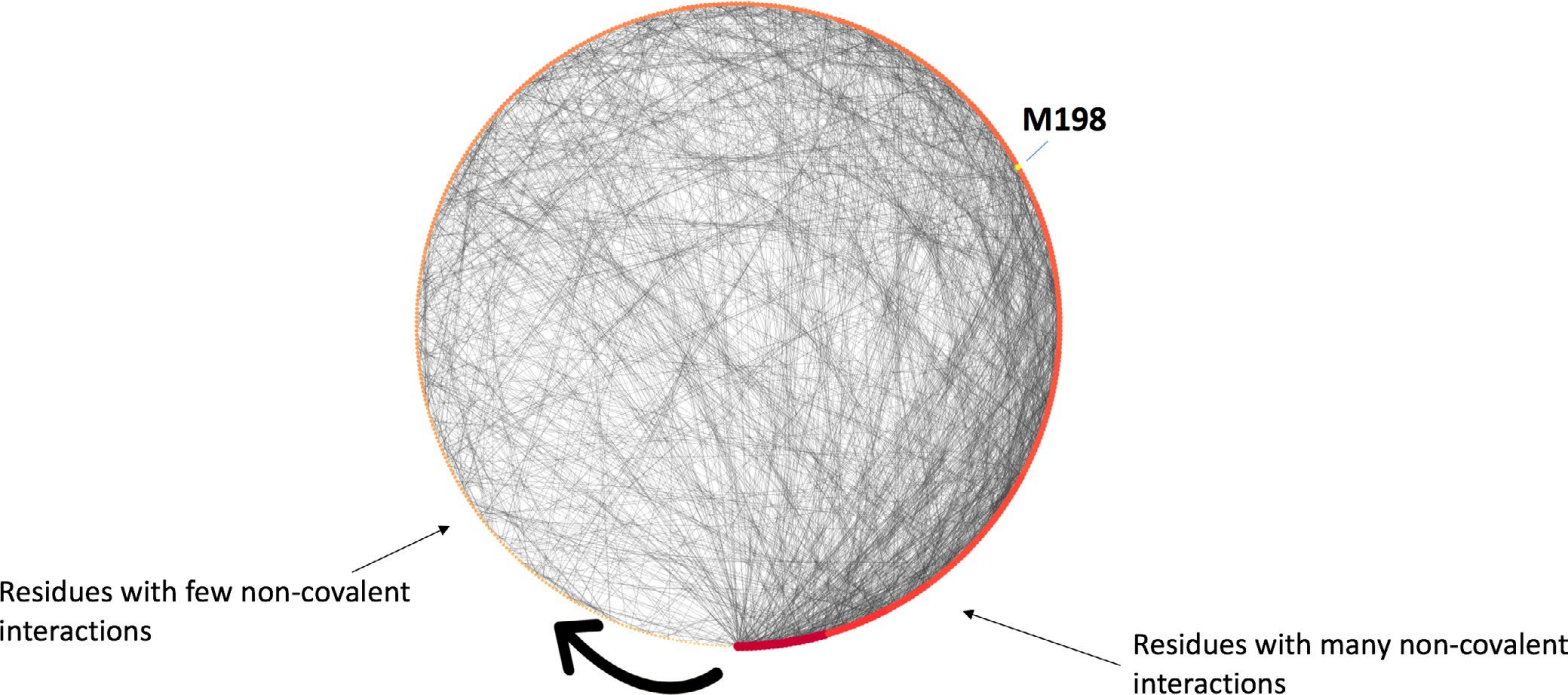
CYP2B6 residues interaction networks. This is a degree sorted (few interactions, small red dots, toward many interactions, large red dot, following the black arrow) circular layout representation of the computed RING network visualized in Cytoscape. The noncovalent interactions are shown as gray lines and each dot on the circle represents a residue (red). Here, we focus only on Met198 (yellow dot) to illustrate the use of such visualization. This residue (node) makes several noncovalent interactions with its surrounding (node degree = 10). The substitution by a smaller residue likely creates a destabilizing hole in the structure

PolyPhen‐2 suggests that this alteration is benign while three structure‐based approaches propose a destabilizing effect (MaestroWeb indicates a possible stabilizing effect) (Table 3). Residue Met198 is relatively large and is essentially hydrophobic while Thr is small and polar. This residue is mainly buried (SA = 26.20%), located far from the catalytic site and tightly packed in a very hydrophobic and aromatic environment (Figure 1). Although Met198 is not fully conserved in our MSA, a hydrophobic residue is always present at this position. It belongs to a possible ligand‐binding pocket as predicted by FTMap (pocket No. 1, Table S2) but it is not present in the predicted channels. The residue makes several noncovalent interactions with its surrounding (node degree = 10; Figure 4). Interactive 3D analysis indicates that the small Thr residue is likely to create a destabilizing hole that could alter proper packing of this region. Overall, the stability investigations and the structural analysis seem in agreement with the experimental data that reports 87% decrease in protein activity level for this substitution (Rotger et al., 2007). Missense3D results suggest that no structural damage is expected due to the substitution while VarSite underlines that the residue is highly conserved in about 190 protein sequences. Yet, the disease propensity score is low with a value of 0.69. For CYP2B6 Met198Thr, most 3D stability investigations and the interactive analysis tend to be in good agreement with the experimental studies. These investigations are not in agreement with the PolyPhen‐2 prediction.

#### 3.2.4 Ile391Asn (allele CYP2B6*15)

PolyPhen‐2 suggests that this alteration is damaging. This is in agreement with the four structure‐based computational packages that indicate a relatively strong destabilizing effect (Table 3). This residue is fully buried (SA = 0.00%) and located in a hydrophobic environment. The residue is essentially conserved or replaced with similar hydrophobic residues in our MSA (Figure S2). This residue is not predicted to be in PPI‐binding surface nor it is part of a predicted ligand‐binding pocket (Table 3). It is situated close to the channel 2f (Figure 2). It has several nonbonded interactions with its surrounding (node degree = 11). Taken together, the hypothesis here is that this amino acid replacement most likely destabilize the protein, in agreement with the in vitro experimental data that shows that this substitution resulted in an almost undetectable enzymatic activity (Lang et al., 2004). Missense3D reports that the substitution will bury a hydrophilic residue while VarSite notes that the residue is highly conserved in 181 protein sequences and that the substitution could impede the protein's function. The disease propensity score is high with a value of 1.99. For CYP2B6 Ile391Asn, most in silico predictions are in good agreement with the experimental studies.

### 3.3 CYP2C9

CYP2C9 metabolizes approximately 15% of the clinically used drugs (Hirota, Eguchi, & Ieiri, 2013; Yiannakopoulou, 2013), including hypoglycemic agents, anticonvulsants, anticoagulants (e.g., warfarin), nonsteroidal anti‐inflammatory (e.g., diclofenac), antihypertensive (e.g., losartan), and diuretic drugs (Isvoran et al., 2017). We selected four clinically important alterations (Arg150His, Ile359Leu, Ile434Phe, and Arg132Gln) associated with altered drug metabolism by >50%. Table 4 shows the results of the in silico analysis for CYP2C9 (PDB ID: http://www.rcsb.org/pdb/search/structidSearch.do?structureId=1OG2, UniProt: http://www.uniprot.org/uniprot/P11712).

**Table 4:** In silico analysis of CYP2C9 (PDB ID: http://www.rcsb.org/pdb/search/structidSearch.do?structureId=1OG2, UniProt: http://www.uniprot.org/uniprot/P11712; GenBank: KF248057.1)

CYP2C9 Amino acid/mutation/alleles	DUET ΔΔG, kcal/mol	PopMusic^a^ ΔΔG, kcal/mol	SAAFEC ΔΔG, kcal/mol	MAESTROweb^b^ ΔΔG, kcal/mol	PolyPhen‐2 Score/mutation prediction	MSA^c^ aa conservation	Involved in predicted ligand‐binding pockets/pocket No. (FTMap)	Involved in known or predicted channels	Involved in predicted PPI sites (meta‐PPISP)	Node degree (RING‐2.0)	Predicted fluctuation value (FlexPred)	Flexibility class^d^ (PredyFlexy)	Decreased metabolic activity or protein expression	References
**Arg132** **R132Q** CYP2C9*33	−0.136 Destabilizing	0.33 Destabilizing SA = 54.9%	−2.112 Destabilizing	1.126 Destabilizing Cpred = 0.756	0.973 Probably damaging	High	No	No	Yes	8	1.563	1	Decreased catalytic activity enzyme toward losartan (in vitro)	Yin et al. (2008)
**Arg150** **R150H** CYP2C9*8	−0.55 Destabilizing	0.15 Destabilizing SA = 60.4%	−5.940 Destabilizing	0.380 Destabilizing Cpred = 0.793	0.071 Benign	High	No	No	No	8	1.359	0	Decrease in enzyme activity (in vitro and in vivo)	Allabi et al. (2005)
**Ile359** **I359L** CYP2C9*3	−0.457 Destabilizing	−0.41 Stabilizing SA = 1.4%	−1.763 Destabilizing	−0.395 Stabilizing Cpred = 0.892	0.002 Benign	High	No	No	No	9	1.144	1	Decreased enzymatic activity (in vitro and in vivo)	Shintani et al. (2001) King et al. (2004)
**Ile434** **I434F** CYP2C9*59	−0.97 Destabilizing	0.70 Destabilizing SA = 22.1%	−0.385 Destabilizing	0.014 Destabilizing Cpred = 0.955	0.969 Probably damaging	High	Yes/1 and 5	No	Yes	6	1.033	1	Greatly decreased enzymatic activity (in vitro and in vivo)	Dai et al. (2015)

a For the program PopMusic solvent accessibility (SA) values are shown (in percent).

b For the program MaestroWeb the confidence estimation Cpred is shown (0.0‐not reliable and 1.0‐highly reliable).

c MSA‐Multiple sequence alignment.

d Flexibility class was determined by the program PredyFlexy (rigid‐0, intermediate‐1, flexible‐2).

Wild‐type residue (bold) and amino acid substitution (Underlined).

Six channels were found for the CYP2C9 protein (PDB ID: http://www.rcsb.org/pdb/search/structidSearch.do?structureId=1OG2) with ChannelsDB (Pravda et al., 2018). None of the studied amino acids is involved in these channels. With regard to the predicted ligand‐binding pockets, 12 pockets were found for CYP2C9 using the FTMap approach (Brenke et al., 2009; Kozakov et al., 2015, 2011; Ngan et al., 2012). From the four investigated mutations, only one amino acid (Ile434) is a part of a predicted binding pocket, this pocket is indeed involved in the binding Heme (predicted pockets 1 and 5, Table S3).

#### 3.3.1 Arg132Gln (allele CYP2C9*33)

PolyPhen‐2 predicts this alteration has most likely a damaging effect (Table 4). This is essentially in agreement with the four structure‐based programs. Arg132 tends to be solvent exposed (SA = 54.88%). The residue is strictly conserved in our MSA (Figure S5) and predicted to be in a relatively rigid region, with several noncovalent interactions with its surrounding (node degree = 8). This residue is not predicted to be in a ligand‐binding pocket nor in a channel but could be in a protein–protein interaction region (Table 4). The residue makes several noncovalent interactions with its surrounding (Table 4). Arg132 is located at the C‐terminus region of an alpha helix (Figure 1). A hydrogen bond is detected between the Arg132 side chain (epsilon N) and the backbone carbonyl of Met129 (a residue located in the helix) that likely stabilizes this region of the protein. Furthermore, alpha helices have a large macroscopic dipole moment (−0.5 charge unit at the C‐terminal). The arginine residue possibly contributes to the stabilization of the helix dipole, a role that the glutamine cannot play. Overall, it seems possible that this amino acid change locally destabilizes the protein and impedes its function. This would be in agreement with the decreased activity observed in vitro (Yin et al., 2008). In addition, Arg132 is predicted to be part of a PPI‐binding region and indeed, it plays an important role in electrostatic interaction with the P450 oxidoreductase (POR) (Lee et al., 2014). The Missense3D report card indicates that the substitution should not damage the protein structure while on the VarSite report it is noted that a Arg to Gln is not dramatic change in term of properties, yet the residue is highly conserved in 181 protein sequences. The disease propensity score is labeled as high with a value of 1.19 and VarSite indicates also that residues equivalent to Arg132 are found at protein–protein interfaces in this family of proteins. For CYP2C9 Arg132Gln, most in silico predictions are in good agreement with the experimental studies.

#### 3.3.2 Arg150His (allele CYP2C9*8)

PolyPhen‐2 labels this change as benign but the structural approaches (more strongly for DUET and SAAFEC) suggest that this amino acid substitution has destabilizing effects (Table 4). Arg150 is essentially solvent exposed (SA = 60.43%), and is involved in several noncovalent interactions with its surrounding (node degree = 8). It is located in the middle of an alpha helix (Figure 1). This residue is strictly conserved in our MSA (Figure S5) and it is not predicted to be in a very flexible region. It is involved in a salt‐bridge network (with Asp143 and Glu147, both also interacting with Arg139, data not shown). Arg150 is not predicted to be located in a binding pocket, in a channel or in a PPI‐binding region. Most likely the shorter histidine residue cannot form the above noted ionic and electrostatic interactions. The substitution could thus destabilize the structure locally. The results obtained with the 3D approaches are in agreement with the experimental data that shows a decreased activity for the variant protein in both in vitro and in vivo systems (Allabi, Gala, & Horsmans, 2005). Missense3D reports that the substitution will not damage the structure (the salt‐bridge network is not seen) while the VarSite report card indicates that a Arg to His may not be dramatic change in term of physicochemical properties, yet the residue is highly conserved in 181 protein sequences. Two natural variants have been identified (Leu and Cys) at position 150. The disease propensity score is high with a value of 1.45. For CYP2C9 Arg150His, most in silico stability prediction methods as well as the interactive structural analysis are in good agreement with the experimental studies. The results of PolyPhen‐2 are not in agreement with these 3D methods nor they support the experimental data.

#### 3.3.3 Ile359Leu (allele CYP2C9*3)

PolyPhen‐2 suggests that this alteration is benign but two structure‐based approaches flag this substitution as possibly destabilizing while PopMusic and MaestroWeb propose a stabilizing effect (Table 4). Ile359 is buried (SA = 1.4%) and located on the C‐terminus of a helical segment (Figure 1). This residue is strictly conserved in our MSA (Figure S5), it is not predicted to be in a highly flexible region. The residue has several noncovalent interactions with its surrounding (node degree = 9). It is not far from the Heme group, yet it is not predicted to be in a ligand‐binding pocket, channels or in a PPI‐binding region (Table 4). Interestingly, interactive structural analysis shows that the Ile359Leu substitution leads to steric clashes in the Heme region although the substitution is very conservative (data not shown). This would seem in agreement with experimental data which indicates a decreased enzymatic activity level for the variant protein (affecting the warfarin dose) (in vitro and in vivo) (King, Khan, Aithal, Kamali, & Daly, 2004; Shintani et al., 2001). Missense3D reports that the substitution will not damage the structure while VarSite mentions that the substitution should be tolerated, although it is also noted that the residue is highly conserved in 182 protein sequences. Two natural variants have been identified (Val and Thr) at position 359. The disease propensity score is low with a value of 0.39 but VarSite still highlights the proximity to the Heme region. For CYP2C9 Ile359Leu, stability prediction methods as well as the interactive structural analysis underline a possible impact on the protein catalytic activity in good agreement with the experimental studies. The results of PolyPhen‐2 are not in agreement with the stability prediction methods, the interactive analysis nor with the experimental data.

#### 3.3.4 Ile434Phe (allele CYP2C9*59)

PolyPhen‐2 suggests that this alteration is most likely damaging with a high score, in agreement with the four structure‐based approaches (more strongly with DUET and PopMusic predictions; Table 4). This residue tends to be buried (SA = 22.05%) and is located near the Heme group. Ile434 makes some noncovalent interactions with its surrounding (node degree = 6; Table 4). The residue is strictly conserved in our MSA (Figure S5) and predicted to be in a binding pocket and in a relatively rigid region. The substitution by a larger aromatic amino acid could damage the folding locally. It should also perturb the binding of the Heme group since it most likely changes the orientation of Cys435, a residue involved in the interaction with ring‐shaped Heme molecule. Moreover, it is also predicted to be next to a PPI‐binding region (not shown) and indeed Ile434 is close to the P450 oxidoreductase (POR)‐binding area (Lee et al., 2014). All these in silico and structural analyses are in good agreement with the greatly decreased enzymatic activity of the variant protein observed in in vitro and in vivo models (Dai et al., 2015). Missense3D reports that the substitution will not damage the structure while the VarSite report card mentions that the substitution should be tolerated, but that the residue is fully conserved in 146 protein sequences. Two natural variants have been identified (Val and Thr) at position 359. The disease propensity score is high with a value of 1.13. VarSite highlights the proximity with the ligand‐binding site and Heme and points to known protein–protein interactions in this protein family. For CYP2C9 Ile434Phe, basically all in silico predictions and the interactive analysis are all in good agreement and help to rationalize the experimental data.

### 3.4 Complement Factor B

For Complement FB (PDB ID: http://www.rcsb.org/pdb/search/structidSearch.do?structureId=2OK5, UniProt ID: http://www.uniprot.org/uniprot/P00751), we investigated the possible molecular effect of four clinically important alterations involved in strengthening and facilitating the formation of an overactive C3 convertase (Table 5). The mutations were identified in patients with aHUS (Marinozzi et al., 2014; Roumenina et al., 2009). For this protein, we predicted putative ligand‐binding pockets with the FTMap. Eleven pockets were identified (Table S4). None of the four amino acids investigated here were found to be in a predicted ligand‐binding pockets.

**Table 5:** In silico analysis of Complement Factor B (PDB ID: http://www.rcsb.org/pdb/search/structidSearch.do?structureId=2OK5, UniProt ID: http://www.uniprot.org/uniprot/P00751; GenBank: X72875.1)

CFB Amino acid/mutation	DUET ΔΔG, kcal/mol	PopMusic^a^ ΔΔG, kcal/mol	SAAFEC ΔΔG, kcal/mol	MAESTROweb^b^ ΔΔG, kcal/mol	PolyPhen‐2 Score/mutation prediction	MSA^c^ aa conservation	Involved in predicted ligand‐binding pockets/pocket No. (FTMap)	Involved in predicted PPI sites (meta‐PPISP)	Node degree (RING‐2.0)	Predicted fluctuation value (FlexPred)	Flexibility class^d^ (PredyFlexy)	Experimental observations	References
**Arg113** **R113W**	−0.472 Destabilizing	0.59 Destabilizing SA = 36.5%	−5.263913 Destabilizing	−0.358 Stabilizing Cpred = 0.884	0.205 Benign	High	No	Yes	10	1.469	1	Normal expression of the recombinant protein. Benign phenotype (in vitro). See text for comments	Marinozzi et al. (2014)
**Arg178** **R178Q**	−1.986 Destabilizing	1.44 Destabilizing SA = 2.88%	−7.039326 Destabilizing	0.023 Destabilizing Cpred = 0.888	1.000 Probably damaging	High	No	No	16	1.176	2	Mutation showed complete lack of functional activity (in vitro)	Marinozzi et al. (2014)
**Asp254** **D254G**	−0.157 Destabilizing	0.09 Destabilizing SA = 38.3%	1.625586 Increase stability	0.162 Destabilizing Cpred = 0.921	0.002 Benign	High	No	Yes	6	1.280	1	Normal expression of the recombinant protein. Gain‐of‐function mutation with enhanced binding to C3b associated with disease (in vitro)	Roumenina et al. (2009)
**Phe261** **F261L**	−1.732 Destabilizing	1.70 Destabilizing SA = 0.34%	0.227907 Increase stability	0.146 Destabilizing Cpred = 0.927	1.000 Probably damaging	High	No	No	14	1.199	0	Mutation with function of strengthening the formation of an overactive C3 convertase leading to aHUS (in vitro)	Marinozzi et al. (2014)

a For the program PopMusic solvent accessibility (SA) values are shown (in percent).

b For the program MaestroWeb confidence estimation Cpred is shown (0.0‐not reliable and 1.0‐highly reliable).

c MSA‐Multiple sequence alignment.

d Flexibility class was determined by the program PredyFlexy (rigid‐0, intermediate‐1, flexible‐2).

Wild‐type residue (bold) and amino acid substitution (Underlined).

#### 3.4.1 Arg113Trp

**Figure 5:**
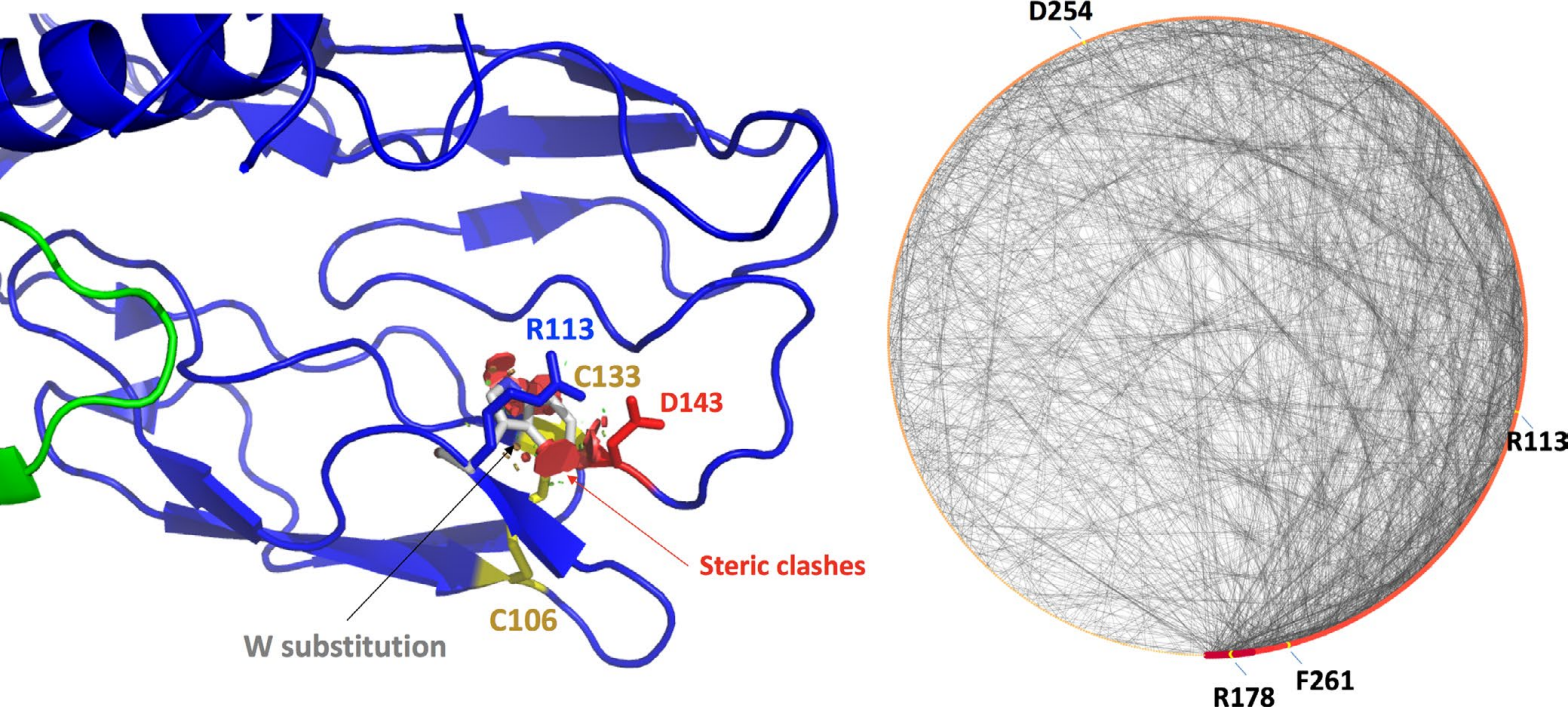
Factor B. The salt‐bridge involving Arg113 and Asp134 (~2.5 Å) is shown. Substitution with a Trp was performed with PyMol and some steric clashes are noticed. A nearby disulfide bond involving Cys106 and Cys133 is also highlighted

PolyPhen‐2 suggests that this alteration is benign but three structure‐based tools indicate that the substitution is destabilizing except for MaestroWeb (Table 5). This residue is highly conserved in the FB sequences from different species (Figure S6) and is not predicted to be in a highly flexible region. It is located at the C‐terminus of a short beta strand (Figure 1), tends to be buried (SA = 36.5%; Table 5) and makes a salt‐bridge with Asp134 (Figure 5). The large W side chain is expected to have local clashes with the surrounding, destroying a salt‐bridge and possibly locally destabilizing the structure of the mutant protein. Arg113 does make several noncovalent interactions with its surrounding (node degree 10, Figure 5), it does not seem to be located in a ligand‐binding pocket but could be part of a protein–protein interaction site. It is difficult to predict if these structural changes will have significant impacts on the 3D structure, because the residue is located nearby the Cys133–Cys106 disulfide bond. The substitution could interfere with the formation of this covalent bond. Alternatively, this covalent bond may compensate the likely destabilizing effect of the substitution. Experimentally, the recombinant CFB was expressed normally without exhibiting a severe phenotype (Marinozzi et al., 2014). The lack of detectable functional consequences is likely related to the localization of the mutation in the Ba part of the molecule, which is cleaved and released in the process of the formation of the active C3 convertase C3bBb. This could also explain the benign phenotype, despite the fact that most predictive tools suggest a local destabilization of the protein domain. Missense3D reports that the substitution will not damage the structure while VarSite mentions that the substitution is important and that the residue is highly conserved in 45 protein sequences. VarSite also points to known protein–protein interactions in this region in this protein family. The disease propensity is high with a value of 1.93. For FB Arg113Trp, basically all 3D in silico predictions and the interactive analysis are all in good agreement with a likely effect on the protein structure. As this region of the protein is cleaved, the phenotype is benign. The 3D tools essentially give a different view as compared to PolyPhen‐2. In this case the PolyPhen‐2 prediction is in good agreement with the experimental data but this also seems to be a lucky prediction. This substitution also highlights the complexity of the biological system. Biophysical studies would be required to fully clarify the impact of the Arg113Trp substitution on the 3D structure.

#### 3.4.2 Arg178Gln

PolyPhen‐2 suggests that this amino acid change is most likely damaging in agreement with all the structure‐based programs (Table 5). This substitution is located in the middle of a short beta strand (Figure 1), is fully conserved in the MSA (Figure S6) and is almost fully buried (SA = 2.88%; Table 5). The residue is present on a complement control protein domain (CCP), in a region that is predicted to be rigid. The residue has numerous noncovalent interactions with its surrounding (node degree = 16, Figure 5) and forms a buried salt‐bridge (predicted to be energetically strong) with Asp422. Arg178 does not seem to be in a ligand‐binding pocket nor involved in a protein–protein interaction site (Table 5). The amino acid change to Gln should alter a strong salt‐bridge and may also perturb the nearby Cys180–Cys140 disulfide bond. The in silico analysis and structural analysis suggest a destabilizing effect, which is in agreement with some experimental data showing that Arg178Gln mutant was expressed partially cleaved and nonfunctional (Marinozzi et al., 2014). Missense3D reports that the substitution will replace a buried charge by an uncharged residue while VarSite mentions that the substitution may or may not be tolerated. The residue is very highly conserved in 45 protein sequences. VarSite gives a relatively high disease propensity score for this substitution with a value of 1.19. For FB Arg178Gln, basically all in silico predictions and the interactive analysis are all in good agreement with a likely effect of the substitution on the protein structure and with the experimental data.

#### 3.4.3 Asp254Gly

PolyPhen‐2 suggests that this change is benign while three structure‐based tools indicate that the substitution is modestly destabilizing except for SAAFEC that predicts a stabilization effect (Table 5). The residue is located in a loop on the von Willebrand domain of CFB (Figure 1), next to a Mg++ binding site, and in a relatively rigid segment. The residue is strictly conserved in the MSA (Figure S6) and partially solvent exposed (SA = 38.3%; Table 5). Its node degree was found to be 6 and this residue makes hydrogen bonds with Gln28 of the nearby complement control protein domain. The substitution could alter these interactions and could increase the flexibility of this loop. The residue is not predicted to be in a ligand‐binding pocket but is expected to be part of a protein–protein binding site. Indeed, experimentally, the Asp254Gly is a gain‐of‐function mutation leading to the formation of an overactive C3 convertase with enhanced binding to C3b (Hourcade, Mitchell, & Oglesby, 1999; Roumenina et al., 2009). This residue is indeed found to be in the binding region for C3b (Marinozzi et al., 2014; Rooijakkers et al., 2009; Roumenina et al., 2009). Interactive analysis suggests a stability change but the main role of this substitution is to affect a PPI‐binding site. The functional consequence, without access to the structure of the C3 convertase (Rooijakkers et al., 2009) could not be predicted by the in silico tools used here, but some hypotheses could be made since the residue is predicted to be in a PPI‐binding region. Interestingly, the type A domain of factor B is similar in structure to the type A domain of the complement receptor and integrin, CR3, in which the residue homologous to Asp254 is also a G. Therefore, the Asp254Gly substitution was studied long before its discovery in aHUS patients and served to delineate the C3b‐binding site (Hourcade et al., 1999) and to create a more stable system, suitable for crystallization (Forneris et al., 2010; Pedersen et al., 2017). Missense3D report card indicates that the substitution should not damage the 3D structure while VarSite mentions that the substitution is significant and that the residue is very highly conserved in 46 protein sequences. VarSite gives a low disease propensity score for this substitution with a value of 1.00 and highlights that the substitution is part of a protein–protein interaction region in this family. For FB Asp254Gly, the in silico predictions and the interactive analysis have difficulties in the prediction of the possible impact of this change. Without experimental data, the key hypothesis that could be made is that the region is involved in protein–protein interactions.

#### 3.4.4 Phe261Leu

PolyPhen‐2 suggests that this substitution is most likely damaging and three structure‐based programs also indicate that the substitution is destabilizing except for SAAFEC that predicts a small stabilizing effect (Table 5). This residue is fully buried (SA = 0.34%) in a tightly packed hydrophobic (node degree = 14) and rigid environment (Table 5). The residue is present in the von Willebrand domain of CFB (Figure 1) and is fully conserved in the MSA (Figure S6). The amino acid change appears to induce steric clashes when investigated interactively and it could indeed be destabilizing. It is not predicted to be in a ligand‐binding pocket nor seems to be directly involved in a protein–protein interaction site (Table 5). It is, however, located nearby a predicted PPI‐binding site. Overall it would seem that this alteration changes the stability in this region of the protein and could affect protein–protein interactions. However, using only the in silico methods, it was not possible to directly predict an increased C3 binding. This mutant was found in aHUS patients and resulted in the formation of a distinct, rapidly cycling C3 convertase, characterized by a faster association but also a faster dissociation rate (Goicoechea de Jorge et al., 2007). Missense3D, reports that the substitution will not damage the 3D structure of the protein while VarSite mentions that the substitution is relatively conservative and that the residue is very highly conserved in 46 protein sequences. VarSite gives a low disease propensity score for this substitution with a value of 0.85. For FB Phe261Leu, the in silico predictions and the interactive analysis have difficulties in the prediction of the possible impact of the mutation. The signal provided by PolyPhen‐2 and more explicitly by the approaches that predict a stability change is that the dynamic of this region of the protein could be changed. As the residue is buried but close to the surface, and close to a predicted PPI site, one could propose a possible impact on protein interactions, but here again, this is a very complex case for the in silico tools.

### 3.5 Factor VIII

The C2 domain (or discoidin domain) of FVIII is known to interact with negatively charged membranes and with other proteins such as von Willebrand factor (VWF) (Liu et al., 2000; Pratt et al., 1999). We investigated the potential molecular effect of two substitutions, Met2238Val (moderate) and Ala2201Pro (mild), located on this domain of FVIII, both associated with hemophilia A (Liu et al., 2000; Pratt et al., 1999; Spiegel et al., 2001; Villoutreix & Miteva, 2016). Table 6 shows the results of the in silico analysis (PDB ID: http://www.rcsb.org/pdb/search/structidSearch.do?structureId=2R7E, UniProt ID: http://www.uniprot.org/uniprot/P00451). Twelve ligand‐binding pockets for the FVIII C2 domain were predicted using the FTMap server (Brenke et al., 2009; Kozakov et al., 2015, 2011; Ngan et al., 2012) (Table S5) and Met2238 and Ala2201 are not directly located in a predicted ligand‐binding pockets. Yet, Met2238 seems next to a predicted PPI binding site while Ala2201 is predicted to be in a PPI‐binding region.

**Table 6:** In silico analysis of blood Factor VIII (FVIII) (PDB ID: http://www.rcsb.org/pdb/search/structidSearch.do?structureId=2R7E, UniProt ID: http://www.uniprot.org/uniprot/P00451; GenBank: NM_000132.3)

FVIII Amino acid/mutation	DUET ΔΔG, kcal/mol	PopMusic^a^ ΔΔG, kcal/mol	SAAFEC ΔΔG, kcal/mol	MAESTROweb^b^ ΔΔG, kcal/mol	PolyPhen‐2 Score/mutation prediction	MSA^c^ aa conservation	Involved in predicted ligand‐binding pockets/pocket No. (FTMap)	Surface exposure (%)^d^	Involved in predicted protein–protein interaction sites (meta‐PPISP)	Node degree (RING‐2.0)	Predicted fluctuation value (FlexPred)	Flexibility class^e^ (PredyFlexy)	Experimental data	References
**Ala2201** **A2201P**	0.378 Stabilizing	0.23 Destabilizing SA = 13.5%	−0.985608 Destabilizing	0.196 Destabilizing Cpred = 0.887	0.988 Probably damaging	High	No	36	Yes	4	0.075	1	Mutation associated with mild hemophilia A The mutant protein domain in vitro seems to have molecular functions similar to the wild type (investigated properties: stability, and binding to the vWF) but membrane binding is damaged	Pratt et al. (1999); Liu et al. (2000). Spiegel et al. (2004)
**Met2238** **M2238V**	−0.503 Destabilizing	0.27 Destabilizing SA = 10.0%	0.553310 Increase Stability	0.248 Destabilizing Cpred = 0.874	0.152 Benign	High	No	11	No but very close to a proposed site	8	1.487	1	Mutation associated with moderate hemophilia A The mutant protein domain in vitro seems to have molecular functions similar to the wild type (investigated properties: stability, membrane binding and binding to the vWF) (in vitro)	Pratt et al. (1999); Liu et al. (2000). Spiegel et al. (2004)

a For the program PopMusic solvent accessibility (SA) values are shown (in percent).

b For the program MaestroWeb the confidence estimation Cpred is shown (0.0‐not reliable and 1.0‐highly reliable).

c MSA‐Multiple sequence alignment.

d Liu et al. (2000).

e Flexibility class was determined by the program PredyFlexy (rigid‐0, intermediate‐1, flexible‐2).

Wild‐type residue (bold) and amino acid substitution (Underlined).

#### 3.5.1 Ala2201Pro

**Figure 6:**
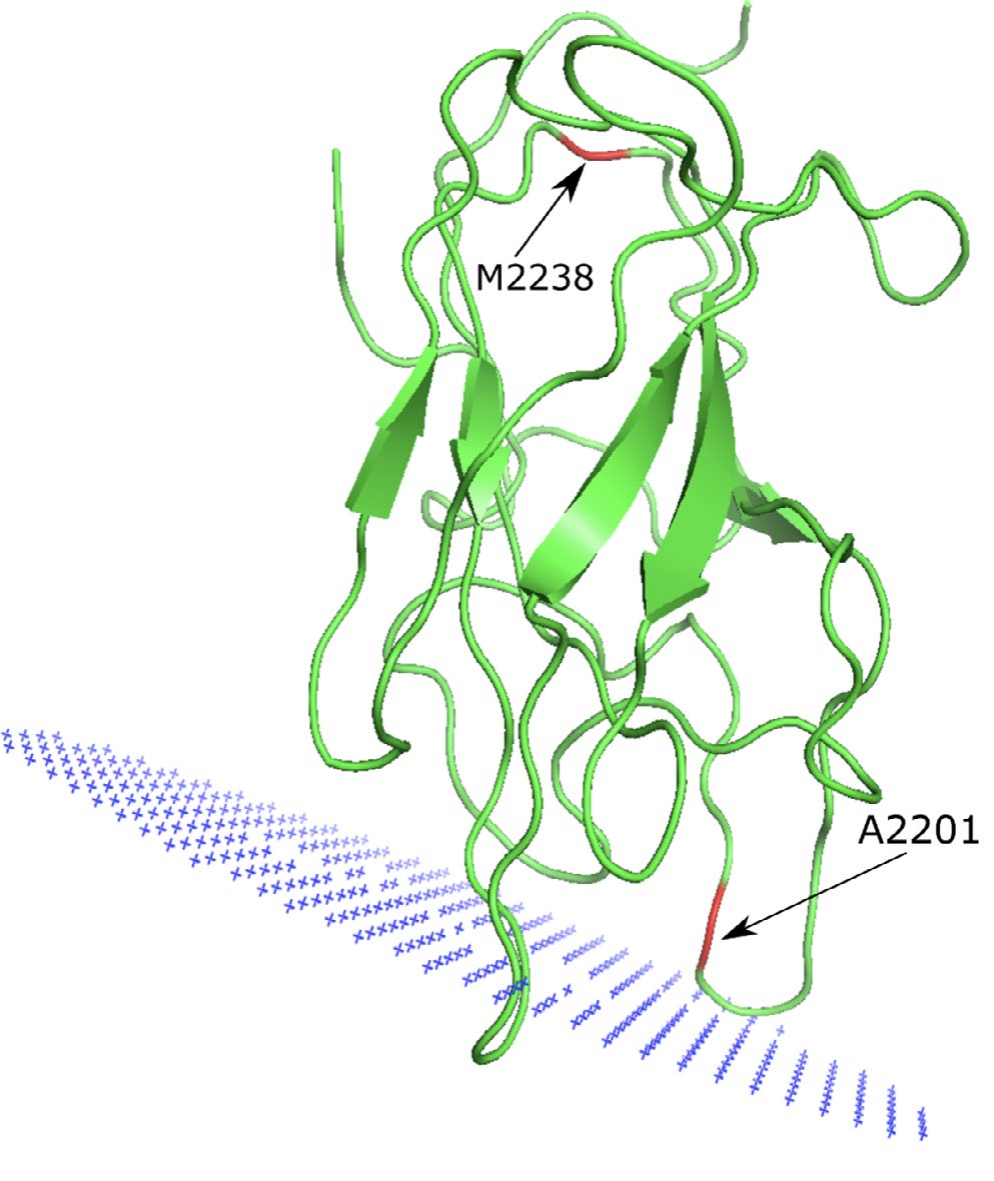
Factor VIII point mutations. The predicted position of the Factor VIII C2 domain on the membrane (in blue), as well as the point mutations investigated in this study are shown

PolyPhen‐2 labels this change as most likely damaging (high score) in agreement with three structure‐based approaches except DUET (Table 6). Ala2201 is well conserved in the sequence (Figure S7; Table 6) with limited noncovalent interactions with its surrounding (node degree = 4) in a region predicted to be relatively rigid. This residue is partially solvent exposed (36%; Table 6) and located on the N‐terminal side of a short beta strand (Figure 1). It is predicted to be both, in a PPI‐binding site (Table 6) and located in a membrane‐binding site as analyzed by the PPM server prediction (Lomize et al., 2012; Figure 6). Interactive structural analysis suggests that its replacement by a Pro should induce a structural change in the nearby loop region of the domain while some steric clashes are also noticed upon introduction of the mutation in the 3D structure. The in silico and interactive structural analysis thus propose a moderate stability change substitution. Experimentally, Spiegel, Murphy, and Stoddard (2004) have shown in vitro that while the substitution does not destabilize significantly the domain, it nevertheless impedes membrane binding, although the interaction with VWF is not affected and the amino acid replacement. It is interesting to note that the in silico analysis predicts this residue to be near a PPI‐binding site. Indeed, it is known that some patients with hemophilia A develop inhibitory antibodies against FVIII. The FVIII C2 domain has been cocrystallized with an antibody prepared from a cell line derived from the memory B‐cell repertoire of a patient with hemophilia A (Spiegel et al., 2001). This Fab fragment interacts with the membrane‐binding loops of the FVIII C2 domain where A2201 is located, thereby impeding the interaction of the cofactor with the membrane. This event most likely occurs when FVIII dissociates from VWF. It is also interesting to note that upon extraction of the Fab fragment from the experimental structure reported by Spiegel et al. (2001) (PDB ID: http://www.rcsb.org/pdb/search/structidSearch.do?structureId=1IQD), it was possible to dock the antibody in a manner that is almost identical to the X‐ray structure (best docking score) with the pyDockWeb server (Jiménez‐García et al., 2013) (data not shown). Further in vivo experimental work has indicated that infusion of FVIII to patients carrying the Ala2201Pro substitution induces a strong immunological reaction and the development of inhibitors against FVIII (Ettinger, James, Kwok, Thompson, & Pratt, 2010) highlighting the complex antigenic changes associated with the 3D structure of the loop harboring amino acid change. Missense3D reports that the substitution will not damage the 3D structure of the protein while VarSite mentions that the residue is very highly conserved in 28 protein sequences. VarSite gives a high disease propensity score for this substitution with a value of 1.58. For FVIII Ala2201Pro, the in silico predictions and the interactive analysis have difficulties in explaining the possible impact of the amino acid substitution on the structure. Some insights come from some 3D prediction tools highlighting a possible PPI‐binding site and protein–membrane interaction site. Clearly, the in silico investigations can help in generating some hypotheses and in designing experiments but cannot fully explain such complex molecular events.

#### 3.5.2 Met2238Val

PolyPhen‐2 suggests that this change is benign and three structure‐based approaches propose a small destabilizing effect with the exception of SAAFEC (Table 6). This residue is essentially buried (SA = 10.0%) with some noncovalent hydrophobic interactions with its surrounding (node degree = 8). M2238 is located in a loop structure, next to the interface with the A domains of FVIII and it is in a relatively rigid area (Figure 1). This residue is well conserved in the sequences or may be replaced, indeed with a valine (Figure S7; Table 6). Interactive structural analysis indicates that the valine side chain can be accommodated in this region of the domain suggesting that if this substitution is involved in bleeding, it may act via unclear mechanism such as the splicing of FVIII or other modifications, as suggested by Spiegel et al. (Spiegel et al., 2004). Missense3D reports that the substitution will not damage the 3D structure of the protein while VarSite mentions that the residue is very highly conserved in 28 protein sequences. VarSite gives a low disease propensity score for this substitution with a value of 0.58. For FVIII Met2238Val, the in silico predictions and the interactive analysis have difficulties in explaining the impact of this amino acid substitution. Taken together, this analysis suggests that more experiments are required to understand if the mutation is responsible for the bleeding phenotype.

### 3.6 Antithrombin

For Antithrombin (AT) (PDB ID: http://www.rcsb.org/pdb/search/structidSearch.do?structureId=2BEH, UniProt ID: http://www.uniprot.org/uniprot/P01008) we investigated the possible molecular effects of four clinically important amino acid substitutions. AT is a major blood coagulation protease inhibitor and becomes an effective inhibitor upon binding to heparin, a negatively charged and highly sulfated polysaccharide that enhances the anticoagulant activity of AT toward its main targets, thrombin, factor IXa, and factor Xa (Huntington, 2011). The crystal structure of antithrombin–heparin and thrombin has been reported several years ago (Li, Johnson, Esmon, & Huntington, 2004). Mutations in the gene encoding AT can cause AT deficiency that predisposes affected individuals to venous and arterial thromboembolism (Ding et al., 2013). According to the phenotype, AT deficiency has been classified into two main categories, type I, characterized by a parallel reduction of the AT antigen and activity levels and type II, defined by a reduction of the AT activity and normal or almost normal antigen levels. The type II AT deficiency has been further subdivided into type IIb with a heparin‐binding defects (type IIHBS) and type IIa with a reactive center loop (RCL) defect and type IIc with pleiotropic defects (Patnaik & Moll, 2008). We initially investigated all the amino acid changes reported in (Luxembourg, D'Souza, Körber, & Seifried, 2015) and then selected two variants with type I deficiency and two variants with type II deficiency. Table 7 shows the results of the prediction for these AT mutants. Using FTMap web server (Brenke et al., 2009; Kozakov et al., 2015, 2011; Ngan et al., 2012) we identified 12 essentially unknown putative ligand‐binding cavities for AT. None of the selected substituted amino acids seem to be located in these predicted pockets (Table S6). Of importance, the binding site for heparin differs from what is known regarding pockets involved in binding small drug‐like molecules. As such, we do not expect to really identify the heparin‐binding site with binding pocket predictors. This is the reason why we used a docking engine to predict the binding of heparin to AT.

**Table 7:** In silico analysis of blood Antithrombin (AT) (PDB ID: http://www.rcsb.org/pdb/search/structidSearch.do?structureId=2BEH, UniProt ID: http://www.uniprot.org/uniprot/P01008; GenBank: NM_000488.3)

AT Amino acid/mutation	DUET ΔΔG, kcal/mol	PopMusic^a^ ΔΔG, kcal/mol	SAAFEC ΔΔG, kcal/mol	MAESTROweb^b^ ΔΔG, kcal/mol	PolyPhen‐2 Score/mutation prediction	MSA^c^ Conservativity level	Involved in predicted ligand binding pockets/pocket No. (FTMap)	Possible interaction with heparin^d^ (ClusPro)	Involved in predicted PPI sites (meta‐PPISP)	Node degree (RING‐2.0)	Predicted fluctuation value (FlexPred)s	Flexibility class^e^ (PredyFlexy)	Experimental data	References
*AT type I*														
**Ser82** **S82R**	−0.439 Destabilizing	1.64 Destabilizing SA = 0.00	−0.509178 Destabilizing	0.025 Destabilizing Cpred = 0.885	1.000 Probably damaging	High	No	No	No	12	1.478	2	AT type I deficiency	Ding et al. (2013)
**Cys95** **C95R**	−0.384 Destabilizing	1.67 Destabilizing SA = 3.79	−2.904263 Destabilizing	1.550 Destabilizing Cpred = 0.785	1.000 Probably damaging	High	No	No	Yes	9	1.016	2	AT type I deficiency	Ding et al. (2013)
*AT type II HBS*														
**Arg13** **R13W**	−0.358 Destabilizing	0.22 Destabilizing SA = 74.69	0.678068 Increase Stability	0.124 Destabilizing Cpred = 0.752	1.000 Probably damaging	High	No	Yes	No	5	2.429	2	AT IIHBS deficiency affecting heparin binding	Olson et al. (2010). Luxembourg et al. (2015)
**Ser116** **S116P**	−0.022 Destabilizing	0.15 Destabilizing SA = 3.64	−0.588801 Destabilizing	−0.117 Stabilizing Cpred = 0.923	0.290 Benign	High	No	No	No	9	1.243	2	AT type IIHBS deficiency affecting heparin binding	Olson et al. (2010). Luxembourg et al. (2015)

a For the program PopMusic solvent accessibility (SA) values are shown (in percent).

b For the program MaestroWeb the confidence estimation Cpred is shown (0.0‐not reliable and 1.0‐highly reliable).

c MSA‐Multiple sequence alignment.

d Heparin–AT docking were carried out with the ClusPro server (see the Methods section).

e Flexibility class was determined by the program PredyFlexy (rigid‐0, intermediate‐1, flexible‐2).

Wild‐type residue (bold) and amino acid substitution (Underlined).

#### 3.6.1 Ser82Arg

PolyPhen‐2 suggests that this substitution is most likely damaging with a high score, in agreement with the four structure‐based approaches where the computed destabilization is relatively strong (Table 7). Ser82 is fully buried (SA = 0.00%) and is located in a hydrophobic and aromatic environment on a helical structure (Figure 1). This residue is fully conserved in our MSA (Figure S8), is not included in a predicted ligand‐binding pockets (Table S6), and cannot be a part of a PPI‐binding site (Table 7). It has several nonbonded interactions with its surrounding (node degree = 12) and packed in a region where some flexibility is required (i.e., located in a region where the RCL inserts when it is cleaved upon interaction with the target proteases). The Ser82Arg substitution generates numerous steric clashes, suggesting it can cause folding problems. The structural analysis is thus in agreement with the experimentally observed type I deficiency. Missense3D reports that the substitution will bury a charged residue while VarSite mentions that the residue is very highly conserved in 108 protein sequences and could impact the function of the protein. Yet VarSite gives a low disease propensity score for this substitution with a value of 0.73. Taken together, all the in silico prediction tools suggest that the AT Ser82Arg substitution could impact the protein structure, in agreement with the experimental data (Ding et al., 2013).

#### 3.6.2 Cys95Arg

PolyPhen‐2 labels this change as most likely damaging with a high score, in agreement with the four structure‐based approaches where the computed destabilization is strong (Table 7). Cys95 is buried (SA = 3.79%) and is located on a moderately flexible loop structure (Figure 1). This residue is fully conserved in the MSA (Figure S8) underlining its importance. Cys95 residue is not predicted to be part of putative ligand‐binding pockets (Table S6) but it could be part of a PPI‐binding site. The residue has several nonbonded interactions with its surrounding (node degree = 10) and forms a disulfide bond with Cys21. This change is predicted to be destabilizing, which is in agreement with the type I phenotype. Missense3D reports that the substitution will disrupt a disulfide bond while VarSite mentions that the residue is very highly conserved in 109 protein sequences and that such substitution is highly unfavorable and will destroy a disulfide bond. VarSite gives a very high disease propensity score for this substitution with a value of 3.27. All in silico predictions suggests that the AT Cys95Arg substitution could impact the protein, in agreement with the experimental data.

#### 3.6.3 Arg13Trp

**Figure 7:**
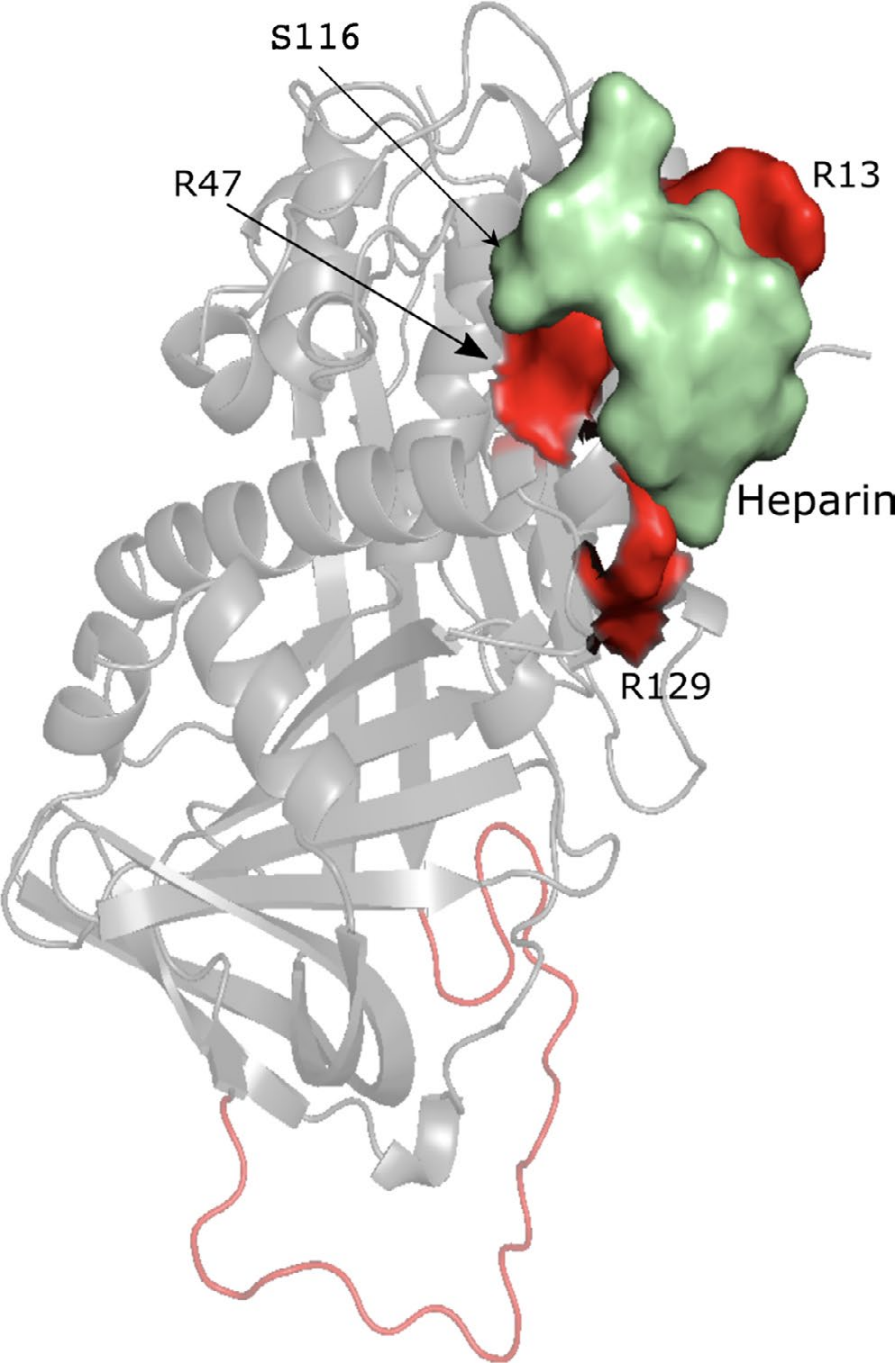
Docking of heparin to AT (PDB ID: http://www.rcsb.org/pdb/search/structidSearch.do?structureId=2BEH) using the ClusPro server. The reactive center loop is in red and the docked heparin is in green

PolyPhen‐2 labels this change as most likely damaging with a high score, in agreement with three structure‐based approaches while SAAFEC indicates a possible increased stability (Table 7). The predicted destabilization is, however, relatively mild. This residue is fully conserved in the AT sequences (Figure S9), it is not involved in a PPI site and not predicted to be in a ligand‐binding pocket (Table S6). Indeed, although it is known experimentally that this zone binds heparin (Johnson et al., 2006), the region does not resemble a typical binding pocket cavity as this surface is essentially flat. Arg13 residue is mainly solvent exposed (SA = 74.69%) and is predicted to be in a relatively flexible region (Figure 1). Docking of heparin to AT is well predicted via the ClusPro server (Kozakov et al., 2017; Mottarella et al., 2014) and the best pose found in silico is very similar to the experimental structure (Li et al., 2004). The docked heparin indicates that Arg13 (as well as Arg47 and Arg129 residues) should be part of the binding site (Figure 7) in agreement with what is known experimentally. Thus, even if the crystal structure of the AT–heparin complex was not available, the in silico approach could have reliably provided insight into the possible molecular impact of the substitution on the structure, function, and heparin binding of the variant protein. The replacement of the Arg by a Trp could indeed alter the binding of heparin to this region of AT without affecting its heparin‐independent inhibitory function, in agreement with a type II HBS phenotype for the mutation. Missense3D proposes that the substitution does not damage the 3D structure while VarSite mentions that the residue is very highly conserved in 92 protein sequences and that such substitution is highly unfavorable. The residue is also found to interact with ligands (i.e., heparin‐like molecules) in several related protein structures. VarSite gives a high disease propensity score for this substitution with a value of 1.93. Taken together, most in silico prediction tools directly or indirectly suggests that the AT Arg13Trp substitution could impact the function protein, in agreement with the experimental data.

#### 3.6.4 Ser116Pro

PolyPhen‐2 suggests that this type IIHBS AT change is benign while three structure‐based approaches propose a moderately destabilizing effect while MaestroWeb indicates a possible stabilizing effect (Table 7). Ser116 residue is fully conserved in the AT sequences (Figure S9), it is not involved in a predicted PPI site and not part of a predicted ligand‐binding pockets (Table S6). The residue is predicted to be in a relatively flexible region and does make several noncovalent interactions with its surrounding (node degree = 9). Ser116 is buried (SA = 3.64%) and located in the N‐terminal side of an alpha helix involved in heparin binding (Figures 1 and 7). Destabilizing effect and local misfolding is possible since the amino acid proline can lead to a change in the orientation of the protein backbone chain. Here again the docking of heparin on the AT surface would have shed light on the potential impact of the amino acid substitution in the absence of an experimental structure of the complex. Taken together, the in silico analyses in 3D provide valuable information on the effect of this amino acid change. This is in agreement with the observed type IIHBS phenotype although the use of PolyPhen‐2 alone here is not sufficient to grasp the possible role of this residue. Missense3D indicates that the substitution will break some hydrogen bonds while the VarSite report card mentions that the residue is very highly conserved in 103 protein sequences and that this residue is also found to interact with a ligand (i.e., heparin‐like molecules) in one experimental protein structure. VarSite gives a high disease propensity score for this substitution with a value of 1.32. Taken together, the 3D in silico prediction tools suggest that the AT Ser116Pro substitution could impact the protein and the function, in agreement with the experimental data. Here PolyPhen‐2 suggested a benign effect.

## 4 CONCLUSION

Numerous in silico prediction packages have been developed to help prioritize potentially deleterious variants. In addition, other in silico tools that have not been developed for the investigation of variants can also be used in an attempt to provide additional insights into the mechanism by which an amino acid substitution may affect the protein structure and/or function.

The present study investigates the possible impact of different types of amino acid changes on the protein structure and/or function using different computational approaches. Some substitutions are easier to analyze while others involve highly complex mechanisms. Among the 20 substitutions investigated here, the experimental data/clinical data are unclear for the FVIII Met2238Val substitution while the possible structural damage is not known for the FB Arg113Trp change. For the other amino acid substitutions, we observed that the PolyPhen‐2 predictions tend to agree with experimental data about 60% of the time and in most cases it is not possible to make suggestion about the possible underlying molecular mechanisms. In silico approaches evaluating stability change (stabilizing or destabilizing), tend to agree most of the time with experimental observations, even though, the molecular mechanisms involved cannot always be explained (e.g., this is for example the case when a substitution favors macromolecular interactions). Conflicting stability predictions among the different tools are known, this can be due to many reasons including scoring function parameters or definition of a threshold to label the substitution as stabilizing or destabilizing. Indeed, it has been shown that for such computations, only the absolute value of the change matters, not the sign (Peng & Alexov, 2016; Petukh, Kucukkal, & Alexov, 2015). Interactive structural analysis can here be used to investigate further the predicted ΔΔG values. When this step is carried out, it is most often possible to clarify the calculation output. When PolyPhen‐2 and 3D stability predictions differ, the residue that is substituted is most often involved in a salt‐bridge in the wild‐type protein structure. The stabilizing or destabilizing nature of a substitution is difficult to estimate with fast computational approaches, but still a stability change can point toward some modification in the dynamics of the system that, if associated with interactive 3D analysis, can provide some insights about the possible impact of the amino acid change. Of importance, when a substitution introduces a titratable residue in the core interior of a protein, the residue may not be charged and pKa computations can provide some insights. Yet, in such circumstance, there is usually an energetic price to pay. In general, in most situations, interactive structural analysis associated with prediction of stability changes and prediction of binding pockets, channels, protein–protein interaction sites or protein–heparin interactions (i.e., prediction of hotspots or prediction making use of docking) provide the users with some rational hypotheses about molecular mechanisms that 2D approaches cannot offer at this time. Definitively, structure‐based programs combined with interactive structural analysis can provide detailed information about the effect of the amino acid change. In this case, the generated hypotheses can in general be rationally translated into assays. 3D approaches complement and even challenge sequence‐based approaches. The combined use of 2D and 3D computational tools is therefore highly recommended, even though, in most clinical genomic centers, the 3D approaches are for the time being largely ignored. Online 3D mapping tools such as VarSite and Missense3D should definitively help biologists with no training in structural biology or structural bioinformatics to rationalize data in 3D. Yet we observe in our dataset that for several substitutions and essentially the ones involving salt‐bridges or salt‐bridge networks, the provided report cards do not report the ionic interactions. For these types of charged substitution, interactive investigations are definitively needed.

## CONFLICT OF INTEREST

The authors have no conflict of interest to declare.

## Data Availability

Data sharing is not applicable to this article as no new missense mutation data were created in this study.
