# Supplementary material for: Analysis of protein missense alterations by combining sequence‐ and structure‐based methods
Source: Mol Genet Genomic Med. 2020 Feb 25;8(4):e1166. doi: 10.1002/mgg3.1166 (PMC7196459; doi:10.1002/mgg3.1166)
Supplement: Supplementary file 1 [file MGG3-8-e1166-s001.docx]

**Analysis of protein missense mutations by combining sequence- and structure-based methods**

Aram Gyulkhandanyan^1,2^, Alireza R. Rezaie^3^, Lubka Roumenina^4,5,6^, Nathalie Lagarde^1,7^, Veronique Fremeaux-Bacchi^4,5,6,8^, Maria A. Miteva^1,9^, Bruno O. Villoutreix^1,10^

^1^ INSERM U973, University Paris Diderot, Laboratory MTi, France

^2^ Laboratory SABNP, University of Evry, INSERM U1204, Université Paris-Saclay, 91025 Evry, France

^3^ Cardiovascular Biology Research Program, Oklahoma Medical Research Foundation and Department of Biochemistry and Molecular Biology, University of Oklahoma Health Sciences Center, Oklahoma City, Oklahoma 73104, USA

^4^INSERM, UMR_S 1138, Centre de Recherche des Cordeliers, F-75006, Paris, France

^5^Sorbonne Universités, F-75006, Paris, France

^6^Université Paris Descartes, Sorbonne Paris Cité, F-75006, Paris, France

^7^ Laboratoire GBCM, EA7528, Conservatoire national des arts et métiers, Paris, France, Hesam Université

^4,6^ Assistance Publique-Hôpitaux de Paris, Hôpital Saint Antoine, Department of Immunology, Paris, France

^8^Assistance Publique – Hôpitaux de Paris, Service d’Immunologie Biologique, Hôpital Européen Georges Pompidou, F-75015, Paris, France

^9^ Inserm U1268 MCTR, CNRS UMR 8038 CiTCoM – Univ. De Paris, Faculté de Pharmacie de Paris, Paris, France

^10^ Université de Lille, INSERM, Institut Pasteur de Lille, U1177 - Drugs and Molecules for living Systems, Lille, France.

**Supplement materials**

**Supplement Figures and Legends**

D438N I386F

HUMAN ---KELDTVI---PETIPHS---

MOUSE ---EELDTVV---PETIPHS---

RAT ---EELDTVI---PETIPHS---

RABBIT ---EELDAVV---PETIPHS---

HORSE ---EELDTVV---PETIPHS---

PIG ---KELDTVI---PETIPHS---

BOVINE ---EELDRVV---PETIPHS---

MACACA ---KELDAVI---PETIPHS---

**Figure S1.** Multiple sequence alignment for CYP1A2 protein for eight different species in the region of the investigated alterations.

Here: Human (Homo sapiens*;* UniProtKB: P05177; GenBank: NM_000761.5), Mouse (Mus musculus; UniProtKB: P00186; GenBank: NM_009993.3), Rat (Rattus norvegicus; UniProtKB: P04799; GenBank: NM_012541.3), Rabbit (Oryctolagus cuniculus; UniProtKB: P00187; GenBank: NM_001171121.1), Horse (Equus caballus; UniProtKB: F6RNE0; GenBank: XM_001493886.5), Pig (Sus scrofa*;* UniProtKB: F1SJ26; GenBank: KM817029.1), Bovine (Bos taurus; UniProtKB: F1MHN9; GenBank: XM_010817139.3), Macaca (Macaca fuscata fuscata*;* UniProtKB: Q4H4C3; GenBank: AB185338.1).

G99E R140Q M198T I391N

HUMAN ---AFSGRGK---MGKRSVE---FLKMLNL----VFLILST---

SHEEP ---AFSGRGK---MGKRSVE---FLRLLEL----VYPILSS---

MACACA ---AFSGRGK---MGKRSVE---FLKILNL----VFPLLST---

CHIMPANZEE ---AFSGRGK---MGKRSVE---FLKMLNL----VFLILST---

PIG ---AFSGRGK---LGKRSVE---FLQLLDL----VYPILTS---

HORSE ---AFSGRGK---MGKRSVE---LRLLDLF----VYPILSS---

DOG ---AFSGRGK---MGKRSVE---LRLMNLF----VFPILHS---

**Figure S2.** Multiple sequence alignment for CYP2B6 protein for eight different species in the region of the investigated alterations.

Here: Human (*Homo sapiens*; UniProtKB: P20813; GenBank: NM_000767.5), Sheep (*Ovis aries*; UniProtKB: W5PBJ5), Macaca (*Macaca fascicularis*; UniProtKB: B8K1S9; GenBank: NM_001287637.1), Chimpanzee (*Pan troglodytes*; UniProtKB: H2R0G3; GenBank: DQ046011.1), Pig (*Sus scrofa*; UniProtKB: F1RHA1), Horse (*Equus caballus*; UniProtKB: L8B938; GenBank: FR848091.1), Dog (*Canis lupus familiaris*; UniProtKB: F1PNK2; GenBank: NM_001006652.1).


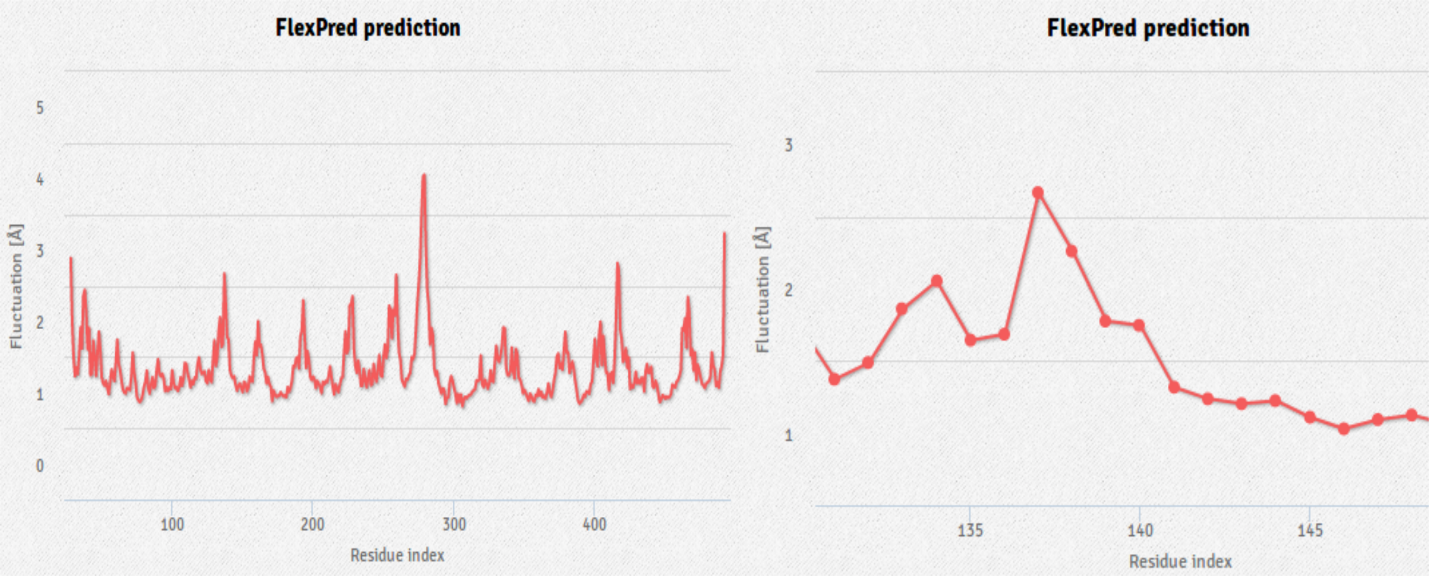


**Figure S3.** Prediction of flexibility for CYP2B6. A zoom on the region of residue Arg140 is shown to the right position. Here the fluctuations of amino acid residues in angstroms (Y axis) along the sequence (X axis).


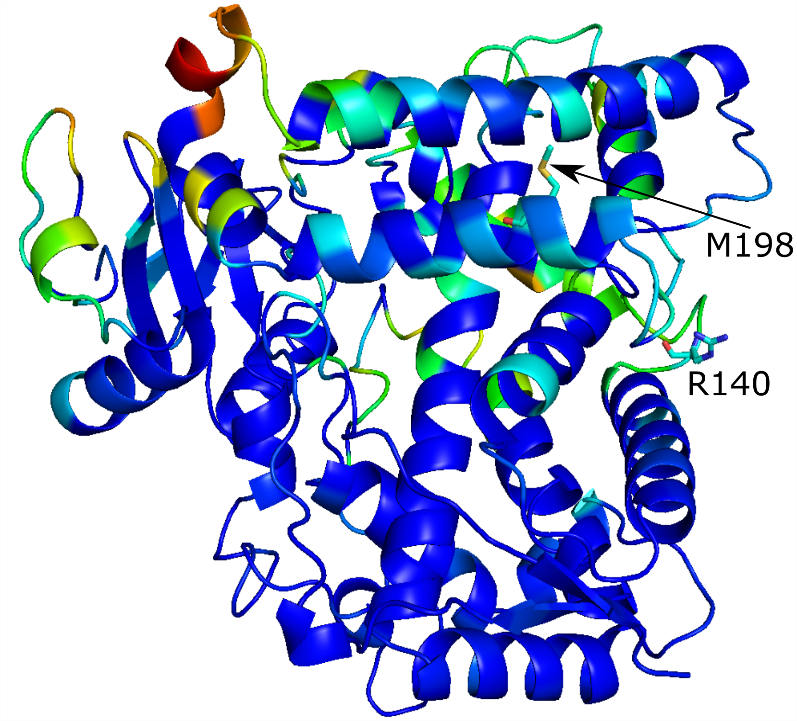


**Figure S4.** CYP2B6 cartoon color coded according to the results of the meta-PPSIP server. High score for PPI sites are colored red while dark blue means unlikely to be involved in PPI. The color spectrum goes from red to dark blue Two amino acids (Arg140 and Met198) are located in a predicted protein-protein interaction site (intermediate score values).

R132Q R150H I359L I434F

HUMAN ---MTLRNLG---EEARCLV---QRYIDLL---GKRICVG---

MOLE RAT ---MTLRNLG---EEARHLA---QRYIDLI---GKRICVG---

MONKEY ---MTLRNLG---EEARCLV---QRYIDLL---GKRICVG---

MACACA ---MTLRNLG---EEARCLV---QRYIDLL---GKRICVG---

GORILLA ---MTLRNLG---EEARCLV---QRYIDLL---GKRICVG---
CHIMPANZEE ---MTLRNLG---EEARCLV---QRYIDLL---GKRICVG---

**Figure S5.** Multiple sequence alignment for CYP2C9 protein for six different species in the region of the investigated alterations.

Here: Human (Homo sapiens; UniProtKB: P11712; GenBank: KF248057.1), Mole Rat (Heterocephalus glaber; UniProtKB: A0A0P6J0T3), Monkey (Cercopithecus sabaeus; UniProtKB: A0A0D9R1I3), Macaca (Rhesus macaque; UniProtKB: F6XQK6; GenBank: NM_001040239.1), Gorilla (Western lowland gorilla; UniProtKB: G3RHD9), Chimpanzee (Pan Troglodytes; UniProtKB: H2Q2B1; GenBank: XM_024346505.1).

R113W R178Q D254G F261L

HUMAN ---YTLRGSA---SQRRTCQ---DGSDSIGASNFTGA---

BOVINE ---YTLRGSA---SEQRTCL---DGSDSVGAHNFTGA---

MOUSE ---YVLRGSA---SQKRKCQ---DGSDSIGSSNFTGA---

ORANGUTAN ---YTLRGSA---SQRRTCQ---DGSDSIGAGNFTGA---

CHIMPANZEE ---YTLRGSA---SQRRTCQ---DGSDSIGASNFTGA---

**Figure S6.** Multiple sequence alignment for Complement Factor B protein for six different species in the region of the investigated alterations.

Here: Human (Homo sapiens; UniProtKB: P00751; GenBank: X72875.1), Bovine (Bos taurus; UniProtKB: P81187; GenBank: NM_001040526.1), Mouse (Mus musculus; UniProtKB: P04186; GenBank: BF116798.1), Orangutan (Bornean orangutan; UniProtKB: Q864W1), Chimpanzee (Chimpanzee; UniProtKB: Q864W0; GenBank: NM_001009169.1).

A2201P M2238V

HUMAN ---ATWS---MKV---

RAT ---SAWP---VKV---

PIG ---ATWS---VKV---

MOUSE ---ATWS---MKV---

DOG ---ATWS---MKV---

**Figure S7.** Multiple sequence alignment for FVIII C2 domain protein for five different species in the region of the investigated alterations.

Here: Human (Homo sapiens*;* UniProtKB: P00451; GenBank: NM_000132.3), Rat (Rattus norvegicus; UniProtKB: D3ZEB1; GenBank: NM_001025152.1), Pig (Sus scrofa*;* UniProtKB: P12263; GenBank: NM_214167.2), Mouse (Mus musculus; UniProtKB: Q06194), Dog (Canis lupus familiaris: UniProtKB: O18806).

S82R C95R

HUMAN ---SPLSIST---LGACNDT---

BOVINE ---SPLSIST---LGACNNT---

SHEEP ---SPLSIST---LGACNNT---

MOUSE ---SPLSIST---LGACNDT---

ORANGUTAN ---SPLSIST---LGACNDT---

MOLE RAT ---SPLSIST---LGACNDT---

**Figure S8.** Multiple sequence alignment for AT type I protein for six different species in the region of the investigated alterations.

Here: Human (Homo sapiens*;* UniProtKB: P01008; GenBank: NM_000488.3), Bovine (Bos taurus; UniProtKB: P41361), Sheep (Ovis aries; UniProtKB: P32262), Mouse (Mus musculus; UniProtKB: P32261), Orangutan (Sumatran orangutan; UniProtKB: Q5R5A3), Mole Rat (Heterocephalus glaber*;* UniProtKB: A0A0P6J376).

R13W S116P

HUMAN ---AKPRDIP---EKTSDQI---

BOVINE ---AKPRDIP---EKTSDQI---

SHEEP ---AKPRDIP---EKTSDQI---

MOUSE ---AKPRDIP---EKTSDQI---

ORANGUTAN ---AKPRDIP---EKTSDQI---

MOLE RAT ---AKPRDIP---EKTSDQI---

**Figure S9.** Multiple sequence alignment for AT type II HBS protein for six different species in the region of the investigated alterations.

Here: Human (Homo sapiens*;* UniProtKB: P01008), Bovine (Bos taurus; UniProtKB: P41361), Sheep (Ovis aries; UniProtKB: P32262), Mouse (Mus musculus; UniProtKB: P32261), Orangutan (Sumatran orangutan; UniProtKB: Q5R5A3), Mole Rat (Heterocephalus glaber*;* UniProtKB: A0A0P6J376).

**Supplement Tables**

| Pockets | Amino acids | Pockets | Amino acids |
| --- | --- | --- | --- |
| Pocket 1 | L98, R180, L382, F384, T385, I386, H388, Q411, L450, F451, G452, M453, G454, K455, R456, R457, C458 | Pocket 8 | R108, T321, F381, L382, F384, T385, I386, Q411, L450, F451, G452, R456, R457, C458 |
| Pocket 2 | R106, L123, T124, W133, R137, D313, I314, A317, K454, R456, R457, C458, I459, G460 | Pocket 9 | R137, Q141, N145, I149, R457, C458, I459, G460, E461, V462, L463, A464 |
| Pocket 3 | T124, F125, F226, V227, G316, A317, G318, D320, T321, T324, L382, I386, L497, T498, K500 | Pocket 10 | L63, Y332, R377, H378, S379, S380, W412, H416, S425, L491, P493, M499 |
| Pocket 4 | I117, T118, S122, L123, T124, F125, F226, F260, N312, D313, G316, A317 | Pocket 11 | I117, T118, F256, N257, F260, N312, G316, A317 |
| Pocket 5 | V54, L57, G58, K59, P61, V227, E228, T229, S231, S232, G233, N234, P235, Y495, G496, L497 | Pocket 12 | L261, L264, Q265, V268, Q269, Y272, F288, Q304, E305, V308, N309, V311, N312 |
| Pocket 6 | L91, Q411, V414, N415, S445, E446, K447, M448, M449, L450, F451, G452, M453, R457, E461, K465 | Pocket 13 | T83, R108, P109, D110, L111, Y112, D237, F238, H388, S389, T390, K404, C405, C406, V407 |
| Pocket 7 | L144, I200, D313, I314, F315, A317, G318, F319, T321, V322, C458, I459, G460, E461, L463, A464 | Pocket 14 | K442, S445, E446, K447, M448, R457, E461, K465 |

**Table S1.** Predicted CYP1A2 ligand binding pockets. Residues located at 5Å around the fragments are listed. The investigated amino acid substitution is colored in red.

| Pockets | Amino acids | Pockets | Amino acids |
| --- | --- | --- | --- |
| Pocket 1 | A176, N177, C180, F184, F188, F195, L196, M198, L199, F202, I241, Y244, I245, L293, F296, T300 | Pocket 11 | L43, M46, D47, R48, R49, G50, L51, S54 |
| Pocket 2 | Y309, F359, S360, D361, L362, S393, H398, L470, T471, P472 | Pocket 12 | S128, V129, M132, C436, L437, G438, E439, G440, I441, A442 |
| Pocket 3 | R85, Y354, Q357, K422, T423, E424, F426, I427, P428, F429, S430, L431, I435,E439, R443 | Pocket 13 | T175, A298, G299, T300, E301, T302, T303, T306, F429, C436, G438, I441, A442, E445 |
| Pocket 4 | F206, F296, F297, A298, G299, T300, E301, T302, T303, L363 | Pocket 14 | R98, L363, G366, V367, H369, L392, P428, F429, S430, L431, R434, I435, C436 |
| Pocket 5 | F206, E301, T302, T305, L362, L363, | Pocket 15 | R98, V113, I114, W121, R125, S294, L295, A298, R 434, I435, C436, L437, G438 |
| Pocket 6 | M132, I178, I179, I182, V183, L264, L295, F296, A298, G299, G438, I441 | Pocket 16 | R49, G50, L51, L52, K53, S54, Q215, P472, C475 |
| Pocket 7 | I101, V104, I114, F115, F206, I209, F297, L363, G366, V367, P368 | Pocket 17 | T302, T303, T306, Q357, L362, L363, P428, F429, S430, A442, L446 |
| Pocket 8 | L52, L56, F59, D361, P364, S393, H397, D406 | Pocket 18 | E218, M365, G366, V367, P368, V388, F389, L390 |
| Pocket 9 | M132, R133, F135, G136, R140, S141, V142, E143, I146, L340, G440, I441, A444 | Pocket 19 | F206, S207, I209, S210, |
| Pocket 10 | E156, M165, D166, P167, T168, F169, L170, F171, S173, Y190, Q191, L196 |  |  |

**Table S2.** Predicted CYP2B6 ligand binding pockets. Residues located at 5Å around the fragments are listed.

| Pockets | Amino acids | Pockets | Amino acids |
| --- | --- | --- | --- |
| Pocket 1 | L87, R97, T301, L362, S365, L366, P367, H368, L391, M426, P427, F428, S429, A430, R433, I434, C435, V436 | Pocket 7 | P174, I178, L293, A297, G298, T299, T302, C435, G437, E438, L440, A441, E444 |
| Pocket 2 | F100, L102, I207, L208,S209, S210, P211, W212, I213, Q214, N217, N474, G475, F476, A477 | Pocket 8 | L102, A103, A106, N107, F114, N204, L208, L233, L234, V237 |
| Pocket 3 | N107, I112, V113, F114, K200, L201, N202, N204, I205, L208, L233, N236, M240, V292, D293, L294, F295, G296, A297, G298, T299, E300 | Pocket 9 | L131, V177, I178, C179, I181, I182, D293, L294, F295, A297, G298, V436, G437, L440 |
| Pocket 4 | R97, G98, I99, F100, P101, L102, A103, E104, F114, L208, I213, Q214, N217, L366, P367, F476 | Pocket 10 | A297, G298, T299, E300, T301, T302, S303, T305, F428, C435, G437, E438, L440, A441, G442, E444 |
| Pocket 5 | R97, I112, V113, W120, R124, L294, A297, L366, R433, I434, C435, V436, G437 | Pocket 11 | N204, I205, E206, L208, S209, E300, A477, V479 |
| Pocket 6 | I112, V113, R124, L131, I178, D293, L294, F295, A297, G298, T299, C435, V436, G437, L440 | Pocket 12 | F100, L 362, T364, S365, L366, P367, L388, F476 |

**Table S3.** Predicted CYP2C9 ligand binding pockets. Residues located at 5Å around the fragments are listed.

| Pockets | Amino acids | Pockets | Amino acids |
| --- | --- | --- | --- |
| Pocket 1 | C78, P79, R118, G124, R125, W126, G128, Q129, T130, P146, I147, G148, E190, P191, S192, C193, Q194, M198 | Pocket 7 | S39, V122, A605, L606, F607, R617, K618, E619, V620, H681 |
| Pocket 2 | Q129, T130, P146, R173, G174, S175, T189, E190, P191, S192, Q194, N415, S419, K421, D422, N423, E424, Q425, H426, V427 | Pocket 8 | P202, Q203, E434, N435, D438, H681, K682, R683, S684, R685 |
| Pocket 3 | V36,C37, P38, S39, G40, F41, Y42, P43 Q47, I76, V122, N123, G124, F197, K431, K604 | Pocket 9 | S39, K604, A605, L606, K618, E619, V620, Y621, I679, V680, H681 |
| Pocket 4 | E34, Y35, V36, C37, P38, S39, G40, F41, Y42, P43, Q47, I76, V122, F197, Y199, K431 | Pocket 10 | R125, R173, D200, T201, Q203, E204, V205, A206, E207, A208, F209, Q425, F428, E458, R460 |
| Pocket 5 | G40, F41, I76, V122, V123, G124, R125, F197, M198, Y199, D200, T201, E204, K431 | Pocket 11 | R25, E34, Y35, V36, C37, Q47, Y199, P407, K431, D432 |
| Pocket 6 | T201, Q203, E204, V205, Q442, V456, W457, E458, H459, K467, R617, H681, S684, F686 |  |  |

**Table S4.** Predicted Complement Factor B ligand binding pockets. Residues located at 5Å around the fragments are listed.

| Pockets | Amino acids | Pockets | Amino acids |
| --- | --- | --- | --- |
| Pocket 1 | L649, S650, V651, T667, L668, T669, L670, H693, N694, S695, D696, F697, L1843, E1844, M1947, G1948, S1949, P1980, G1981, V1982 | Pocket 7 | L649, L668, H693, S695, D696, F697, R700, S1839, L1843, E1844, K1845, V1847, M1947, G1948, S1949, N1950 |
| Pocket 2 | R279, S289, T292, L294, M662, D666, F671, K1967, M1973, L1975, Y1976, N1977, L1978, Y1979, V1982, E1984 | Pocket 8 | C153, L154, T155, Y156, R279, F293, L294, T294, K1972, M1973, A1974, L1975 |
| Pocket 3 | W1835, A1836, Y1837, S1838, I1852, G1853, P1854, L1855, F1879, L1945, S1946, M1947 | Pocket 9 | Q283, L286, E287, I288, S289, I291, T292, L294, F671, P672, F673, N1977, Y1979 |
| Pocket 4 | L668, S1784, Y1786, S1787, S1788, A1836, Y1837, S1838, S1839, D1840, V1841, L1843, V1847, G1853, M1947 | Pocket 10 | D150, P151, L154, T155, V278, R279, F293, L294, T295, A296 |
| Pocket 5 | T667, L668, T669, S1788, W1835, A1836, L1945, S1946, M1947, G1981, V1982, F1983 | Pocket 11 | R279, V1965, R1966, K1967, M1973, Y1976, E1984 |
| Pocket 6 | F270, L271, E272, G273, H274, T275, A284, H478, G479, I480, T481, H497, K499, D519 | Pocket 12 | A1836, Y1837, S1838, L1843, V1847, L1851, I1852, G1853, M1947 |

**Table S5.** Predicted Factor VIII C2 domain ligand binding pockets. Residues located at 5Å around the fragments are listed.

| Pockets | Amino acids | Pockets | Amino acids |
| --- | --- | --- | --- |
| Pocket 1 | F62, H65, L66, A67, S69, K70, G328, F329, S330, L331, K332, E333, Q334, L335, D337 | Pocket 7 | L130, Y131, R132, K133, A134, N135, K136, A137, S138, K139, L140, V141 |
| Pocket 2 | I7, T85, N127, C128, Y131, R132, S142, N144, E163, L164, V165, Y166, G167 | Pocket 8 | Y240, K241, A242, L285, P286, K287, P288, E289, K290, S291, L292, A293, R406, P407, F408 |
| Pocket 3 | D6, I7, C8, N127, R132, S162, E136, L164, V165, Y166, G167 | Pocket 9 | P16, N18, L92, G93, N155, T157, Y158, I161, G353, I354 |
| Pocket 4 | M89, Y131, S142, A143, N144, R145, Y166, G167, A168, W189, K193 | Pocket 10 | R129, L130, K133, A134, D278, E414, P416, L417 |
| Pocket 5 | P41, E42, A43, T44, W49, S52, Q305, R413, E414, V415, P416, L417, N418, T419 | Pocket 11 | K226, F258, R259, E271, L272, P273, D277, T280, V415 |
| Pocket 6 | Y131, R132, L140, V141, S142, G167, K193 | Pocket 12 | N75, R322, F323, R324, I325, N428, P429, C430, V431 |

**Table S6.** Predicted AT type I and AT type II HBS ligand binding pockets. Residues located at 5Å around the fragments are listed.
